# Identification and Characterization of Hundreds of Potent and Selective Inhibitors of *Trypanosoma brucei* Growth from a Kinase-Targeted Library Screening Campaign

**DOI:** 10.1371/journal.pntd.0003253

**Published:** 2014-10-23

**Authors:** Rosario Diaz, Sandra A. Luengo-Arratta, João D. Seixas, Emanuele Amata, William Devine, Carlos Cordon-Obras, Domingo I. Rojas-Barros, Elena Jimenez, Fatima Ortega, Sabrinia Crouch, Gonzalo Colmenarejo, Jose Maria Fiandor, Jose Julio Martin, Manuela Berlanga, Silvia Gonzalez, Pilar Manzano, Miguel Navarro, Michael P. Pollastri

**Affiliations:** 1 Instituto de Parasitología y Biomedicina "López-Neyra" Consejo Superior de Investigaciones Cientificas, Granada, Spain; 2 Department of Chemistry and Chemical Biology and Center for Drug Discovery, Northeastern University, Boston, Massachusetts, United States of America; 3 Tres Cantos Medicines Development Campus, DDW and CIB, GlaxoSmithKline, Tres Cantos, Spain; McGill University, Canada

## Abstract

In the interest of identification of new kinase-targeting chemotypes for target and pathway analysis and drug discovery in *Trypanosomal brucei,* a high-throughput screen of 42,444 focused inhibitors from the GlaxoSmithKline screening collection was performed against parasite cell cultures and counter-screened against human hepatocarcinoma (HepG2) cells. In this way, we have identified 797 sub-micromolar inhibitors of *T. brucei* growth that are at least 100-fold selective over HepG2 cells. Importantly, 242 of these hit compounds acted rapidly in inhibiting cellular growth, 137 showed rapid cidality. A variety of *in silico* and *in vitro* physicochemical and drug metabolism properties were assessed, and human kinase selectivity data were obtained, and, based on these data, we prioritized three compounds for pharmacokinetic assessment and demonstrated parasitological cure of a murine bloodstream infection of *T. brucei rhodesiense* with one of these compounds (NEU-1053). This work represents a successful implementation of a unique industrial-academic collaboration model aimed at identification of high quality inhibitors that will provide the parasitology community with chemical matter that can be utilized to develop kinase-targeting tool compounds. Furthermore these results are expected to provide rich starting points for discovery of kinase-targeting tool compounds for *T. brucei,* and new HAT therapeutics discovery programs.

## Author Summary

Human African trypanosomiasis, or sleeping sickness, affects 10,000 patients annually, yet current drugs for this disease are poor, with high toxicity and inconvenient dosing requirements. *Trypanosoma brucei,* the parasite that causes sleeping sickness, is sensitive to a class of compounds called kinase inhibitors, and our project was aimed at identifying kinase-targeting compounds that rapidly and irreversibly inhibit parasite growth. This was accomplished by high-throughput screening of over 42,000 compounds, which resulted in identification of 797 potent inhibitors of parasite growth that are non-toxic to human cells. These inhibitors were studied for the speed of their effects and reversibility of growth inhibition, and were grouped on the basis of chemical structure similarity. One compound was shown to cure mice from a bloodstream of infection of *T. brucei*. These compounds can now be utilized by the research community as starting points for new drug discovery, and also as tool compounds for understanding the function of kinases in *T. brucei*.

## Introduction

Human African trypanosomiasis (HAT) is a parasitic infection that affects 10,000 patients annually [1]. Current therapies for HAT have significant issues of toxicity, inconvenient dosing regimens, and emerging resistance. While there are new therapeutic options currently being evaluated in the drug development pipeline, such as SCYX–7158 [2], nifurtimox-eflornithine combination therapy [3], and fexinidazole [4,5], there remains a need for back-up approaches to new drugs for this disease.

In recent years, in response to repeated calls for new drugs from the World Health Organization and clear guidelines for new drug specifications for HAT [6], drug discovery efforts have increased worldwide for this otherwise neglected disease. The acute lack of financial incentive for undertaking the costly drug discovery process is increasingly being addressed by a combination of public research funding, as well as philanthropic and industrial contributions. The OpenLab Foundation was established in 2010 as a means to provide financial support to drug discovery efforts performed at GlaxoSmithKline (GSK) in collaboration with investigators outside the company who have identified new approaches to fighting tuberculosis [7], and tropical diseases such as HAT, malaria [8,9], Chagas disease, and leishmaniasis. Indeed, in recent years large sets of screening data for compounds tested against the pathogens causing these diseases have emerged from this unique combination of industrial, philanthropic, and non-industrial collaborators [7,8].

One powerful approach to discovery of new drugs for HAT has been directed at repurposing established knowledge about classes of molecular targets that the pathogen holds in common with humans, recognizing that the huge body of historical knowledge around homologous targets could be quickly redirected to inhibiting pathogen growth [10]. These target families include phosphodiesterases [11], histone deacetylases [12], and kinases [13,14,15,16,17].

*T. brucei* expresses 176 kinases [18,19], an observation that has driven our efforts to identify classes of kinase inhibitors that can be useful for discovery of new parasite growth inhibitors. For example, we recently reported that the human PI3K and mTOR inhibitor NVP-BEZ235, a Phase III clinical candidate for solid tumors, is highly potent against trypanosomes in culture and *in vivo* [14]. Other PI3K and mTOR inhibitors were also shown to be potent lead compounds for killing trypanosomatid parasites. Recognizing that we had only tested a very small fraction of the kinase inhibitor chemotype space, and that the kinome of *T. brucei*, *Leishmania,* and *T. cruzi* have been analyzed and found to have moderate similarity to human kinases [18], we hypothesized that testing a much wider set of human kinase inhibitor chemotypes should allow identification of a wide range of starting points for HAT therapeutics.

Over the last few decades, drug discovery efforts have focused on discovery of inhibitors for specific molecular targets (enzymes, receptors), though it is now becoming apparent that combining whole-cell activity data with molecular mechanism of action information is, in fact, the most productive path forward to new discovery [20]. Further, since little detail is known about the functions of the *T. brucei* kinome, the discovery of putative kinase-targeting inhibitors that potently inhibit cell growth represent an opportunity not only for new leads for HAT, but also new tool compounds for elucidation of kinase function in the pathogen.

**Figure 1:**
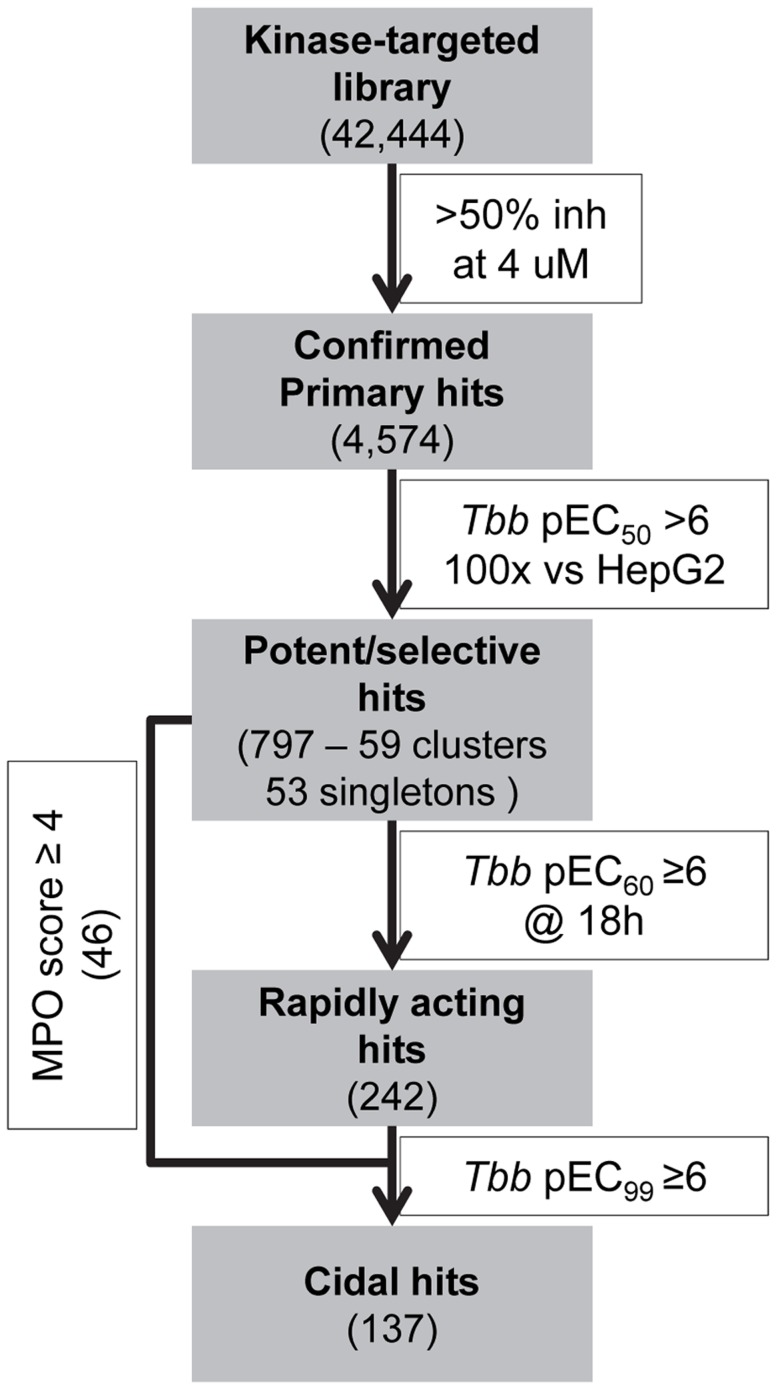
The project assay cascade.

With this in mind, we now report the assessment of 42,444 kinase-targeted compounds in a high-throughput, cell-based *T. brucei* growth assay, followed by evaluation of cellular selectivity, characterization of the rate and reversibility of action, coupled with a range of predictive and experimental determinations of drug-like properties that can inform prioritization for future drug discovery efforts for HAT. To our knowledge, this represents the largest kinase-targeted HTS against *T. brucei*, resulting in discovery of 797 validated *T. brucei* growth inhibitors, grouped into 59 clusters (plus 53 singleton compounds), intended to prompt new studies of mechanism of action and further pursuit for drug optimization for HAT. We further describe the prioritization of 46 clusters based on potency, rate-of-action, cidality, and predicted central nervous system (CNS) exposure. The progression through the HTS process and follow-up experiments is shown in **Figure 1**.

## Materials and Methods

### Cellular assays

#### Single concentration screening assay (primary HTS campaign)

Greiner sterile black clear bottom 384-well assay plates were pre-dispensing with 200 nL of compound in each well, from master plates at 1 mM (100% DMSO) containing selection from the GSK compound collection. Controls of 0% response (control 1, 200 nL of 100%DMSO) and 100% response (control 2, 200 nL of 1 mM pentamidine) were included in each assay plate in columns 6 and 18, respectively. Plates containing 200 nL of 100% DMSO were included randomly in the assay, to assess quality through all process. To detect growth inhibition, *T. brucei brucei* parasites (Lister 427 strain) in log phase were diluted to a working concentration of 2,500 cells/mL in prewarmed HMI-9 medium, and gently stirred until dispensation. 50 µL of culture were dispensed in compound-stamped black, clear-bottom 384-well Greiner microplates using a Multidrop Combi Reagent Dispenser (Thermo Scientific), to give a final solvent concentration of 0.4% DMSO. Plates were covered with lid and cells were incubated for 70 hours at 37°C and 5% CO_2_. After this period, 10 µL of 200 µM resazurin solution in prewarmed HMI-9 were added to each well, and plates were allowed to incubate 2 hours more prior fluorescence reading in a Wallac EnVision Multilabel Plate Reader (Perkin-Elmer). Raw fluorescent data from the Envision plate reader were uploaded into GSK HTS database (ActivityBase). Activity of each well was normalized as a percentage of inhibition per-plate basis using the following equation:
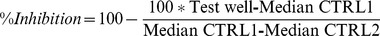



Where control 1 represents wells from the same plate containing 0.4%1DMSO (0% inhibition, 100% grown control, n = 16), and control 2 represents wells from the same plate treated with Pentamidine (100% inhibition, 0% grown, n = 16). A Z′ value greater than 0.4 was required for plate validation during the quality control process.

#### Dose response assay

For dose-response experiments, serial compound dilutions of selected hits from the primary campaign were plotted against compound concentration. Dose response master plates were prepared by 3-fold dilutions (starting with 10 mM DMSO stock solutions), providing 11 concentrations. From these master plates, 200 nL per well was stamped in final assay plates, including the same controls describe in the primary single shot assay. Compounds were dispensed as serial dilution curve format, with 11 points at 3-fold dilution and 40 µM as starting top concentration (n = 2). Growth inhibition assay was performed as described in previous assay.

A 4-parameter equation describing a sigmoidal dose-response curve was then fitted with an adjustable baseline using ActivityBase XE Runner software. Fitting of dose-response curves and EC50 determination were normalized as percentage of inhibition based on controls. The curve-fit model was based on a 4-parameter equation:
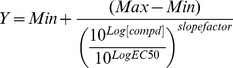



#### Cytotoxicity assay

This assay was used as selectivity assay, and selected hits from the primary were test in dose response concentration against HepG2 in order to identify the level of host cell cytotoxicity. Dose response starting at 10 mM and with 3-fold dilutions for 11 points, were made in 1,536-well master plates. 50 nL per well from them were stamped in final assay plates. The human biological samples were sourced ethically and their research use was in accord with the terms of informed consent.

Log-phase HepG2 cells were removed from a T-175 TC flask using Cell Dispersion Medium and dispersed by repeated pipetting. Cell density was adjusted to 60,000 cells/mL as working concentration in prewarmed Eagle's MEM medium. Seeding density was be checked to ensure that new monolayers are not more than ∼ 50% confluent at the time of seeding (typically 3,000 cells per well), before completing preparation of plates. 5 µL of culture were dispensed in compound-stamped TC treated, Greiner white 1,536-well plates using a Multidrop Combi Reagent Dispenser (Thermo Scientific), to a final concentration of 2.5% DMSO. Cells were incubated for 48 hours at 37°C and 5%CO_2_. Viability was determined by Cell Titer Glo Kit (Promega) according to manufacturer's instructions: briefly, reconstituted Cell Titer Buffer was equilibrated to room temperature prior its use and 5 µL per well were dispensed with a Multidrop Combi Reagent Dispenser. Contents were mixed on a plate orbital shaker and incubated for 10 min at room temperature, to allow the signal to stabilize before luminescence reading on ViewLux Plate Reader (Perkin-Elmer).

Raw Luminescence data from the ViewLux reader were uploaded into GSK HTS database (ActivityBase). Activity of each well was normalized as a percentage of inhibition per-plate basis using the following equation:


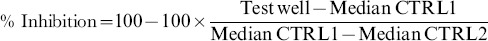


Where control 1 represents wells from the same plate containing 1% DMSO (0% inhibition, 100% grown control, n = 128), and control 2 represents wells from the same plate treated with Digitoxin (100% inhibition, 0% grown, n = 128). A Z′ value greater than 0.4 was required for plate validation during the quality control process.

As previously described for the primary assay, a 4-parameter equation describing a sigmoidal dose-response curve was then fitted with an adjustable baseline using ActvityBase XE Runner software.

#### Rate of action assays

Serial compound dilution of selected hits from single concentration screening assay, starting at 10 mM and with 3-fold dilutions for 11 points, were made in master plates. From these master plates, 30 nL per well were stamped in final assay plates Greiner 1,536-well Solid White, including controls of 0% response (DMSO) and 100% response (40 µM pentamidine): compound final assay top concentration was 40 µM. 8 µL of *T. brucei* culture in log phase were dispensed on assay plates using a Multidrop Combi Reagent Dispenser (Thermo Scientific), after adjusting cell density according to the different incubation time points described in the text; 4 sets of compound plates were arranged to assay in order to be sequentially stopped at their correspondent time point, and plates containing DMSO were included randomly in the assay, to assess quality through all process. Plates were incubated at 37°C and 5% CO_2_ for the indicated time points; incubation was stopped by addition of 2 µL of Cell Titer Glo reagent (Promega), and after shaking the plates were incubated at room temperature for 30 min, to allow the signal to settle. Plate luminescence was read on a ViewLux multiplate reader (Perkin Elmer), and raw data were processed and analyzed with ActivityBase software. As previously described percentage of inhibition was calculated for each concentration based on controls. A 4-parameter equation was used to fit the dose-response curves and pEC_50_ determination.

#### Reversibility assays

Serial compound dilution of selected hits from rate of action assay, starting at 10 mM and with 3-fold dilutions for 11 points, were made in master plates. 100 nL per well from master plates were stamped in final assay black, clear-bottom 384-well Greiner microplates, including controls of 0% response (DMSO) and 100% response (2 µM Pentamidine): the final assay concentration was 20 µM. Plates containing DMSO were included randomly in the assay, to assess quality through all process. *T. brucei* cells in log phase were diluted to a working cell density of 3,000 cells/mL, and culture was dispensed in assay plates using a Multidrop Combi Reagent Dispenser (Thermo Scientific), while gently stirred; after dispensation, plates were covered with lid and incubated at 37°C and 5%CO_2_. After 18 hours incubation, plates were centrifuged in a centrifuge (Beckman Coulter) for 5 min at 500 g, to sediment the cells, supernatant was aspirated with a Cybio-Well dispenser (Cybio), and 40 µL/well of fresh prewarmed HMI-9 were added: this cycle was performed three times. After the last cycle, 10 µL/well of cells suspension were aspirated by Cybio-Well equipment and dispensed over 40 µL of fresh prewarmed HMI-9 in “recovery plates”: calculated maximal residual concentration was 32 nM. The recovery plates were incubated for 70 hours at 37°C and 5%CO_2_: after this period, 10 µL of 200 µM resazurin solution in prewarmed HMI-9 were added to each well, and plates were allowed to incubate 2 hours more prior fluorescence reading in a Wallac EnVision Multilabel Plate Reader (Perkin-Elmer). Assay stadistical quality was determined by Z' score and validated data set were analyzed with ActivityBase software. As previous described percentage of inhibition was calculate for each concentration based on controls. A 4-parameter equation was used to fit the dose-response curves and pEC_50_ determination. The potential effect of residual drug remaining after washes was evaluated by performing the same protocol with standard trypanocidal drugs (such as suramin and diminazene) and negligible effect was observed.

### ADME and physicochemical properties assays

#### CLND kinetic solubility assay

5 µL of 10 mM DMSO stock solution was diluted to 100 µL with pH 7.4 phosphate buffered saline, equilibrated for 1 hour at room temperature, filtered through Millipore Multiscreen_HTS_-PCF filter plates (MSSL BPC). The filtrate was quantified by suitably calibrated flow injection Chemi-Luminescent Nitrogen Detection [21]. The standard error of the CLND solubility determination is ±30 µM, the upper limit of the solubility is 500 µM when working from 10 mM DMSO stock solution.

#### GSK in-house artificial membrane permeability assay

A 1.8% lipid (phosphatidyl choline, egg) in 1% cholesterol decane solution was applied to a Millicell 96-well, 0.4 µm, PCF culture plate. 250 µL and 100 µL 50 mM phosphate buffer pH 7.4 with 0.5% encapsin was applied to the donor and receiver compartments, respectively. 2.5 L of a 10 mM stock solution of compound in DMSO was added to the donor compartment. The assay was incubated at RT for 3 hours. Samples from both donor and receiver compartments were analyzed by HPLC with UV detection at 215 and 254 nm and permeability was calculated. The permeability (logP_app_) measures how fast molecules pass through the lipid membrane is expressed in nm/s.

#### ChromlogD assay

The Chromatographic Hydrophobicity Index (CHI)[22] values were measured using reversed phase HPLC column (Luna C18 (2), Phenomenex, UK) with a fast acetonitrile gradient at starting mobile phase of pH = 7.4. CHI values are derived directly from the gradient retention times by using a calibration line obtained for standard compounds. The CHI value approximates to the volume % organic concentration when the compound elutes. CHI is linearly transformed into ChromlogD by least-square fitting of experimental CHI values to calculated ClogP values for over 20,000 research compounds using the following formula: ChromlogD  =  0.0857CHI-2.00. The average error of the assay is ±3 CHI unit or ±0.25 ChromlogD.

#### Protein binding assay

Chemically bonded Human Serum Albumin (HSA) and Alpha-1-acidglycoprotein HPLC stationary phases (Chiral Technologies, France) were used for measuring compounds' binding to plasma proteins, applying linear gradient elution up to 30% iso-propanol. The run time was 6 min, including the re-equilibration of the stationary phases with the 50 mM pH7.4 ammonium acetate buffer. The obtained gradient retention times were standardised using a calibration set of mixtures as described in the references [23]. The average standard error of the assay depends on the binding strength and kinetics of the compounds. It ranges from ±5% in the medium binding range which reduces to 0.1% at binding above 99% with fast kinetics.

#### Phospholipid binding assay (IAM)

Compounds binding to immobilized artificial membrane (IAM) has been measured using commercially available IAM PC DD (Regis Analytical, West Lafayette, USA) HPLC column. Applying acetonitrile gradient up to 70% the gradient retention times of the compounds were converted to Chromatographic Hydrophobicity Indices (CHI IAM) using a calibration set of compounds. The CHI IAM values then were converted to the logarithmic retention factors using the following formula, obtained from the correlation of isocratic and gradient retention time [24]:

log k IAM  =  0.046*CHI IAM+0.42,

#### Cytochrome P450 (CYP450)

The P450 inhibition profile of the inhibitors was determined as previously described [25].

### *In vivo* experiments

#### Ethics statement

*Efficacy experiments:* Guidelines of the European Convention for the Protection of Vertebrate Animals used for Experimental and other Scientific Purposes (CETS #123) were applied to maintain and care of mice used in this work. The animal experimental protocol CEEA2013/MNC/2 used for African trypanosome studies was reviewed and approved by the Ethical Committee of the Instituto de Parasitología y Biomedicina “López Neyra” and the Ethical Committee of the Spanish National Research Council (CSIC). *Pharmacokinetic experiments:* All experiments were approved by the Diseases of the Developing World (DDW-GSK) ethical committee, and performed under protocol number AP1925v2. All animal studies were ethically reviewed and carried out in accordance with European Directive 2010/63/EU, Spanish legislation RD53/2013, and the GSK Policy on the Care, Welfare and Treatment of Animals.

#### Mouse pharmacokinetics

For pharmacokinetic studies NMRI female mice of 20–25 g weight were used. Experimental compounds were administered by the following routes at a volume of 10 mL/kg (n = 3 mice per route): compound NEU-1200, intravenous (iv) bolus at 1 mg/kg and intraperitoneal (ip) bolus at 5 mg/kg. Compounds NEU-1207 and NEU-1053, iv bolus at 1 mg/kg, ip bolus at 5 mg/kg and by oral gavage (po) at 5 mg/kg.

All mice received treatment in the fed state. Drugs were administered as solution in the following vehicles: NEU-1200, Saline (pH adjusted to 4.8); NEU-1207, 20% Encapsin/5% DMSO in saline solution; NEU-1053, 20% Encapsin/5% DMSO/7.5% PEG400 in saline solution.

Peripheral total blood was the compartment chosen for the establishment of compound concentrations. Aliquots of 20 µL of blood was taken from the lateral tail vein for each mouse at the following time points post-dose: for iv and ip route, 5, 15 and 30 min, 1, 2, 4, 6 and 8 h; for po route, 15, 30 and 45 min, 1, 2, 4, 6 and 8 h.

LC-MS was used as the analytical method of choice for the establishment of compound concentration in blood with a sensitivity of LLQ  =  1–5 ng/ml in 20 µL of blood. The non-compartmental data analysis (NCA) was performed with WinNonlin Phoenix 6.3 (Pharsight, Certara L.P) and supplementary analysis was performed with GraphPad Prism 6 (GraphPad Software, Inc). The pharmacokinetic parameters are shown in tabular format in the Supporting Information, **Table S7**.

#### *In vivo* efficacy experiments

Stock solutions (30 µg/mL) of NEU-1053 were prepared in DMSO, with the final concentration ≤6.3% DMSO, a concentration that did not exhibit any toxicity for the parasite or mice. For treatment, NEU-1053 was prepared in PBS (Sigma-Aldrich) and then heated at 50°C for 10 min to solubilize.

Female NMRI mice were obtained from Charles River Laboratories and were kept in a conventional room at 20–24°C with a 12/12-h light/dark cycle. The animals were provided with sterilized water and were fed *ad libitum*. Infection was performed by i.p. injection of 10^4^ bloodstream forms of *T. b. rhodesiense* (EATRO3 ETat1.2 TREU164) *or T. b. brucei* (Lister 427) strain in 0.2 mL TDB glucose. Three days later, infected animals with confirmed parasitemia were divided into two equal groups: Control (infected mice, and treated with the vehicle DMSO (6.3% in PBS)); and Drug-treated (infected mice treated with 2 doses of 10 mg of drug per kg of body weight per day (20 mg/kg/d)). Four mice in each group were used in this assay. Drug-treated mice received a 0.2 mL ip injection at the 3^rd^ day from infection during 4 consecutive days. After a 4 day hiatus, drug was administered for additional 4 days.

Parasitemia was individually checked by direct microscopic counting of parasites in a Neubauer chamber using 2 µL of blood from infected mice tail, diluted in 500 µL of TDB glucose. In order to increase the sensitivity of parasite detection, the collected blood was diluted in 20 mL HMI-9 medium, distributed in a 96 well plate (Thermo Scientific) and was incubated at 37°C and 5% CO_2_. In addition, a 200× dilution of the initial 20 mL was also monitored for parasite growth. This strategy allowed us an improved parasitemia detection (500 parasites/mL of blood). Mortality was examined daily until 90 days post-infection and expressed as a percentage of cumulative survival.

## Results

### Compound library selection

A kinase inhibitor subset was selected to be screened in the growth assay using three approaches. First, compounds from the GSK compound screening collection that possess>70% Tanimoto similarity to the previously-reported active PI3K/mTOR inhibitor chemotypes [14] were selected (2,979 compounds). Second, we included the GSK Published Kinase Inhibitor Set (367 compounds) [26]. Third, we included the subset of 39,098 kinase inhibitor compounds from the wider screening collection to create a full 42,444 member kinase-targeted library.

### High-throughput screening

**Figure 2:**
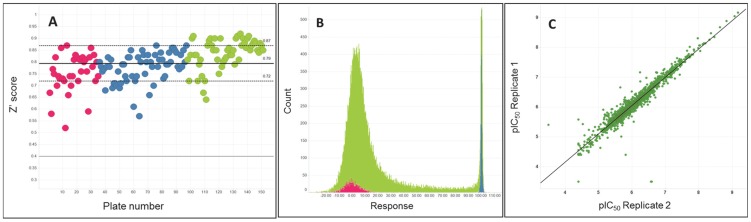
(A) Ź trend over single shot campaign duration. (B) Distribution of %inhibition response in single shot assay. Plates assayed were run on 3 different days, identified by different colors in plots A and B. (C) Correlation of 4,574 compounds in dose response assay (n = 2).

A high-throughput screen (HTS) was developed using a *T. b. brucei* (Lister 427 strain) whole-cell assay based on a widely-validated resazurin viability test [27] in HTS format. During assay development, final experimental conditions were assessed in terms of DMSO and compound concentration, inoculum density and resazurin-dependent fluorescence, and validated with standard trypanocidal drugs and kinase inhibitors [14,28]. The selected compounds from the GSK collection were tested at 4 µM concentration in a single point assay to test their growth inhibition in a log-phase culture of *T. b. brucei* using the optimized assay conditions. The HTS was performed in 384-well format, with a Z′ robust value mean of 0.78, a mean signal-to-background> 5 and a throughput of ∼17,600 compounds per day [29]. Representative HTS performance metrics are available in **Figure 2**, and additional details are described in the Supporting Information.

Approximately 15% of the original set (6368 out of 42,444) displayed more than 50% growth inhibition at 4 µM drug concentration in the HTS close to the robust statistical cut-off (3 SD). These compounds were subjected to a confirmatory single concentration assay, and tested in duplicate sets, leading to 4,574 compounds to be advanced to *T. brucei* and HepG2 cell dose-response assays. We selected a relatively short incubation time for the HepG2 cell assay (24-48 hours) to allow identification of compounds with acute host cell toxicity. Taking these assays together, we found 797 compounds to have EC_50_ values ≤1 µM against *T. brucei* cells, with at least 100-fold selectivity over HepG2 cells.

### Analysis of compound properties

**Figure 3:**
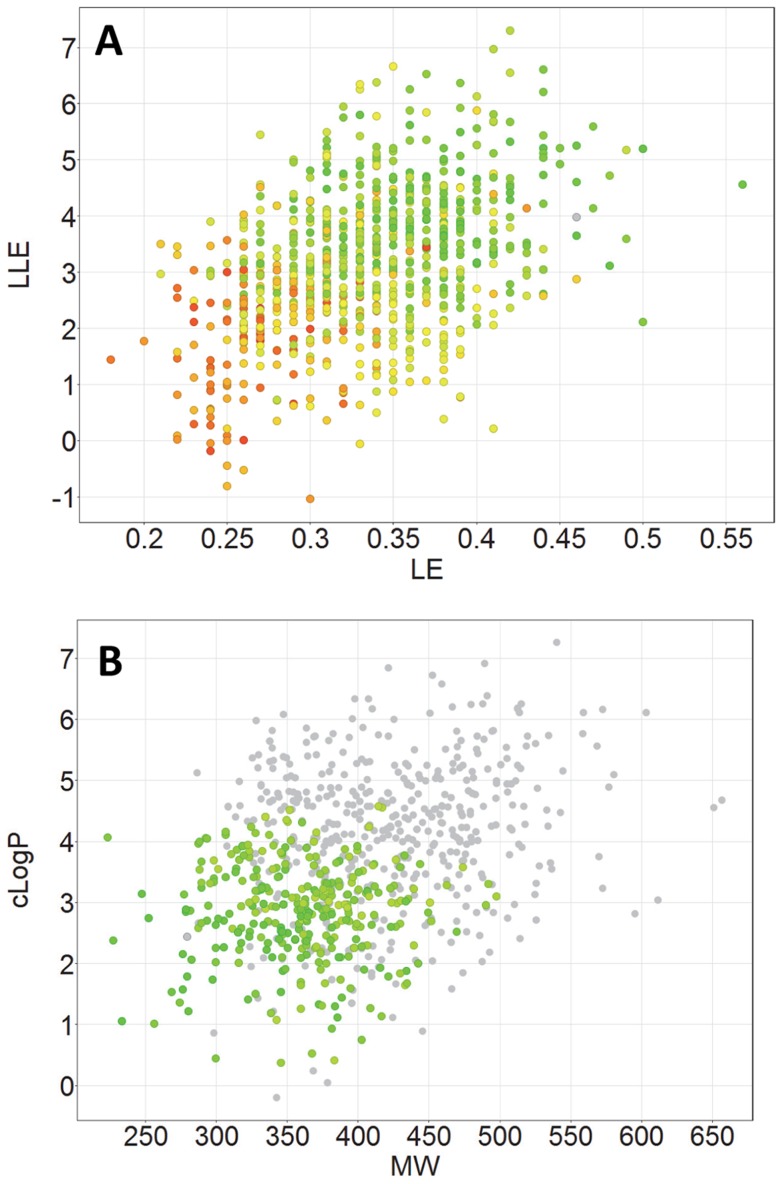
Representation of 797 hit compounds. (a) Plot of LLE vs LE, color coded based on MPO score (min = 1.1, Red; max = 5.75, Green). (b) plot of molecular weight vs cLogP. Compounds with MPO score ≥4 are colored green.

Ligand efficiency (LE) is a characterization of a compound's efficiency on a per-heavy-atom basis. Calculated by 1.37*pEC_50_ divided by the number of heavy atoms, a value of LE ≥0.3 is typically accepted as a good starting point for optimization efforts [30]. Although ligand efficiencies come from a thermodynamic analysis of target-ligand interactions, we use them here as a score or descriptor to prioritize compounds based on their balance of activity vs molecular size and lipophilicity. LE is most commonly utilized in analysis of target-based assay results, but, as demonstrated in a recent antimalarial program, it can be utilized to normalize potency for molecular size in cell assays as well [31]. Of the 797 hit compounds, 538 (or 68%) show an LE>0.3 (**Figure 3A**). The cLogP and molecular weight of the hits are plotted in **Figure 3B**, color coded for compounds that are predicted to permeate the CNS based on a predictive model (*vide infra*). Lipophilic ligand efficiency (LLE, pEC_50_-cLogP) has recently been shown to be reliable and meaningful metric of inhibitor quality [32,33], with a targeted LLE≥4 [34]. Of the hit compounds, 200 (25%) show an LLE value of at least 4, and 242 compounds (30% of the hits) have a LLE value between 3–4.

**Figure 4:**
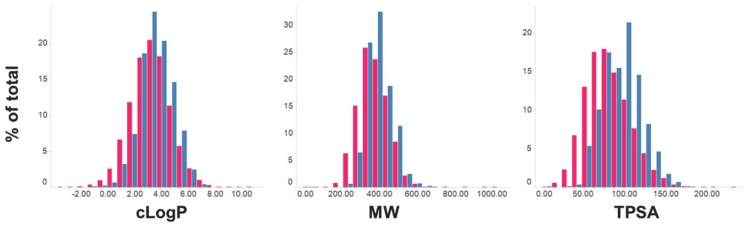
Histogram distributions of cLogP, MW and TPSA for (a) the whole HTS set (42,444 compounds, red), and (b) the 797 potent and specific compounds (pEC_50_>6 and 100-fold specificity, blue).

Computed properties of the hit compounds, as a comparison to the overall screening set are shown in **Figure 4** and are summarized in **Table 1**. The distributions of the potent and specific compounds are narrower, compared to the overall screening set, with mean values slightly increased when compared to the whole HTS set.

**Table 1:** Summary of average properties of the overall screening set and specific compounds.

	Mean values		
	HTS Set (n = 46,688)	Hits (n = 797)	Δ
**cLogP**	3.24	3.55	0.31
**MW**	372.28	395.12	22.84
**TPSA**	81.27	96.53	15.26

Noting that new HAT therapeutics must be centrally-acting, we then computationally characterized the hit compounds for predicted central nervous system (CNS) activity. We applied the method recently disclosed by Wager *et al.* that utilizes commonly computed properties (cLogP, cLogD, molecular weight, topological polar surface area (TPSA), hydrogen bond donors, and pKa) to predict each compound's likelihood of CNS activity [35]. In this model, preferred ranges for each of the properties above are identified, compounds are scored based on compliance with each of those property ranges, and these properties scores are summed to obtain the CNS multiparameter optimization (MPO) score. Using this method, compounds with a CNS MPO score of 4 or higher is predicted to be centrally acting. (We note that one difference between our MPO calculation and that performed by Wager *et al.* is the method for computation of pKa. We utilized the pKa computation from ChemAxon, whereas previous work utilized that available from ACD/Labs). **Figure 3B** shows a plot of cLogP versus molecular weight of the 797 potent and selective compounds; 329 compounds (41% of the total) have a MPO score ≥4 (green), and are predicted to have a high propensity for CNS activity.

### Rate-of-action and cidal/static studies

In order to identify the most effective anti-trypanosomal agent, we sought compounds that would rapidly (≤18 hours) and irreversibly kill *T. brucei* cells. While the initial assays above were carried out at 72 h using resazurin as a redox indicator of cellular viability and density, we performed a different cell viability dose-response assay at shortened compound-treatment periods (6, 12, 18 and 24 h) [2]. For these experiments we utilized ATP content as an indicator of living cells, which achieves a lower level of detection than the resazurin assay [36]. This allowed us to use lower cell densities to quantify cell death as signal decay. We note that the different readout had no effect upon compound potency at 72 hours.

Since cell density remains almost invariable for 6 hours of incubation, the biological activity of compounds near this time point predominantly reflects induction of cell death. However, at incubation times>24 hours, when cell density has significantly increased, the action of compounds almost exclusively reflects growth inhibition. Thus, assessment of cell density at intermediate incubation times (i.e. 12 and 18 hours) can allow us to discern between compounds that act via cell killing or via growth inhibition. Compounds that do not quickly kill the parasite or arrest its growth will appear inactive at 6 hours in the ATP content assay and active at 72 hours in the resazurin-based one. Conversely, compounds with a rapid action will already exhibit their effects at short (6 hour) incubation times, with increasing potency with longer incubation times.

**Figure 5:**
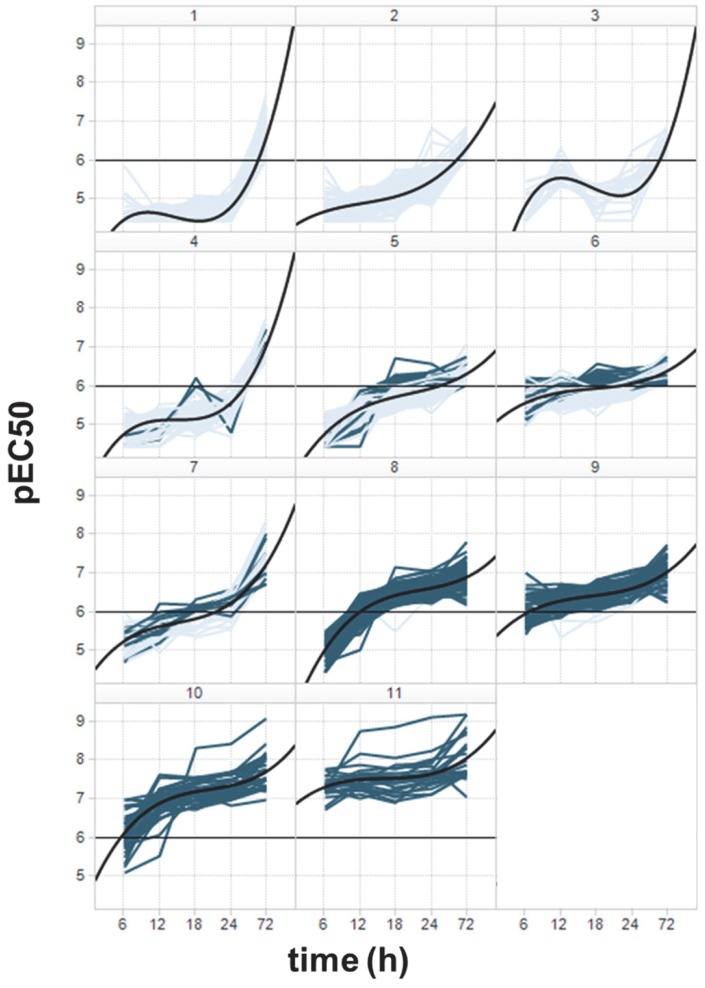
Rate-of-action curves for 797 potent *T. brucei* growth inhibitors, shape-clustered. Average rate-of-action within a given cluster is shown as a polynomial fit to the curves (black). Curves for rapidly acting compounds (pEC_50_>6 at 18 h) are depicted in dark blue.

With this analysis in mind, the pEC_50_ values for each compound were plotted as a function of time, generating rate-of-action curves, which were shape-clustered into 11 clusters by using a K-means algorithm with Euclidean distances (**Figure 5**). The average shape of the curves in each cluster (the black curves in **Figure 5**) was delineated by fitting a third degree polynomial to all the points in each cluster, allowing classification of the curve clusters, such that different clusters display different rate of action behavior. For instance, slow-acting compounds (pEC_50_≥6 only after 72 hours) are contained mainly in clusters 1, 4 and 7. Fast-acting compounds are contained mainly in clusters 8–11, where pEC_50_≥6 was achieved as early as 12 hours of incubation time. The remaining clusters show intermediate behaviors, with a moderate but consistent increase in potency with increasing incubation time. Thus, 242 compounds showed a pEC_50_≥6 by 18 hours of incubation, were classified “rapidly acting” compounds and are shown as dark blue curves in Figure 5.

We selected these rapidly-acting molecules for progression into compound washout studies to determine their cidal/static properties, and also tested 46 slower-acting compounds that possessed computed properties consistent with good CNS penetration (CNS MPO score≥4). Dose-response assays were performed as above, but, after 18 hours of incubation, drugs were washed from the cell cultures. The resazurin cell viability assay was performed 72 h after drug removal to determine the extent to which the cells were able to resume replication and growth. We defined the minimum trypanocidal concentration (MTC, EC_99_) as the compound concentration that abolishes 99% of growth recovery when compared with DMSO controls. We define “cidal” compounds to be those with pEC_99_>6 after 18 hours of incubation.

**Figure 6:**
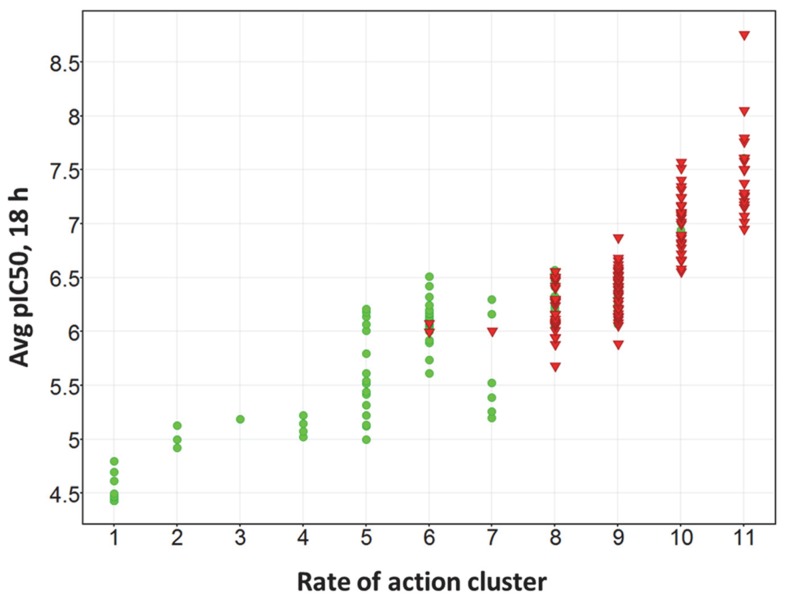
Scatter plot showing the average pEC_50_ at the 18 hour time point for the 242 compounds tested in the cidality assay. Compounds are colored on the basis of reversibility behavior (cidal = red; static = green).

By this definition, 56% (or 137) of these rapidly acting compounds are cidal. Within this set, 24 compounds achieved pEC_99_ values>7. Further analysis also revealed that 96% of the slow acting compounds that were included in these cidality assays were static in behavior. Stated differently, fast acting rate-of-action clusters (clusters 8–11) contain compounds that are predominantly cidal, while other clusters are predominantly static (**Figure 6**).

### Selection and computation of properties for prioritization

At this point we performed a series of computations that would help further prioritize these compounds in combination with the CNS MPO scores described above.

We decided that a multi-factorial prioritization of screening hits was warranted with a focus on specific properties that we decided would be most valuable for selection of the most promising chemotypes for future hit-to-lead optimization. We developed a Composite Score that consisted of potency, ligand efficiency, lipophilic efficiency, rate of action, and the cidal/static nature of the inhibitor. These criteria were selected on the basis of the desired properties for HAT therapeutic candidates (prioritizing potent, fast-acting, cidal, and predicted brain-penetrant compounds). The relative scoring was designed with this in mind, and based on our collective drug discovery experience. Under this scheme, the maximal score was 16, calculated as shown in **Table 2**. For example, compounds with pEC_50_≥8 scored 3 points, those between 7–8, 2 points, and between 6–7, 1 point. Compounds that were rapidly acting (pEC_50_>6 at 18 hours) received 2 points, and those whose pEC_99_ of 6 or greater received 2 points. We used a similar approach for scoring predicted CNS activity (MPO Scores), and compound efficiencies (LE, LLE). Points were totaled for each compound, providing the Composite Score. **Table S5** in the Supporting Information shows the distribution of the HTS hits in each of the Composite Scoring bins.

**Table 2:** Compound composite scoring schema.

	Score			
	3	2	1	0
pEC_50_	≥8	8>x≥7	7>x≥6	
Rate of action^a^		Fast		Slow
MPO Score	≥5	3≤x<5	1≤x<3	<1
Cidal^b^		Yes		No
LE	≥0.4	0.4>x≥0.3	<0.3	
LLE	≥6	6>x≥5	5>x≥4	<4

a “Fast” is defined as pEC_50_≥6 at 18 h.

b “Cidal” is defined as pEC_99_≥6 in the reversibility experiments. Only compounds that were rapidly acting and/or had an MPO score ≥4 were tested in the cidality assay; compounds not tested in the cidal assay are assigned a score of 0.

### Compound clustering

**Figure 7:**
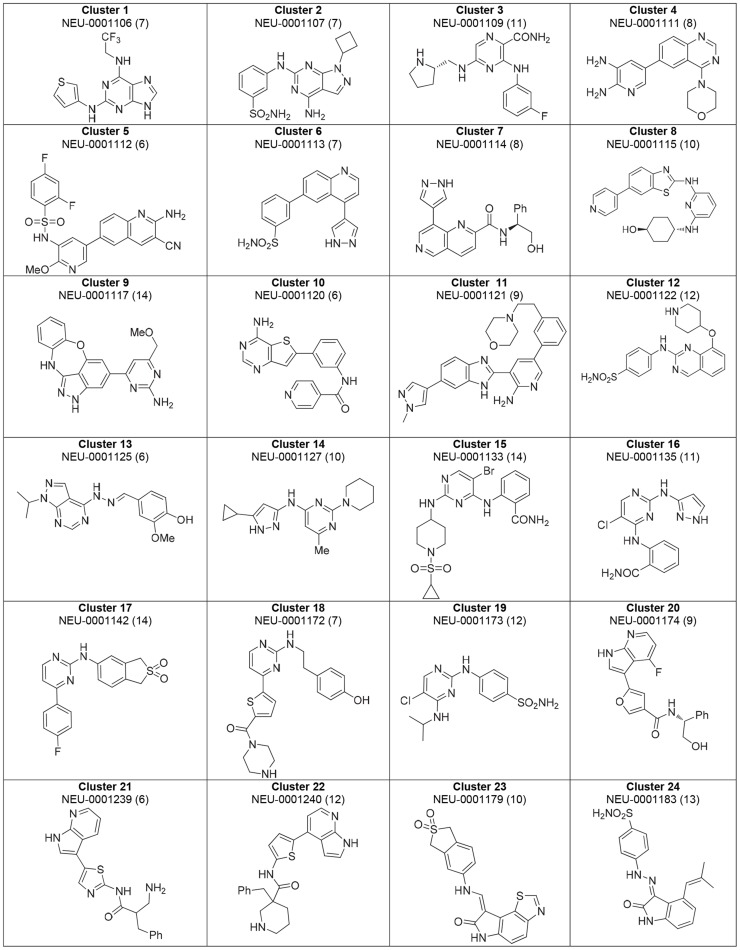
Highest-ranking cluster representatives for clusters 1-24. Each compound's composite score is shown in parentheses.

**Figure 8:**
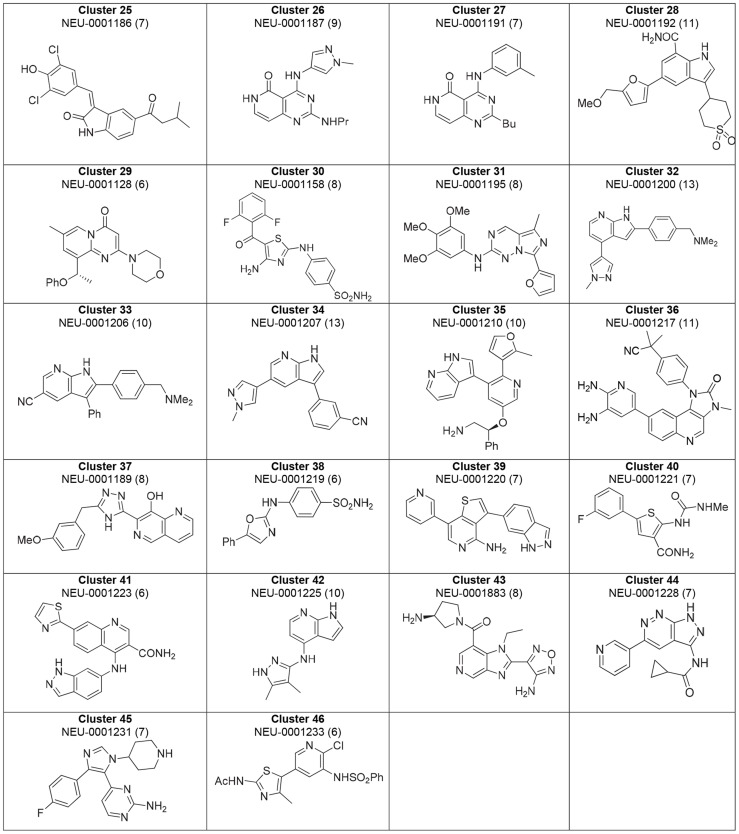
Highest-ranking cluster representatives for clusters 25-46. (Clusters 47-59 only contain compounds with composite scores <6). Each compound's composite score is shown in parentheses.

**Figure 9:**
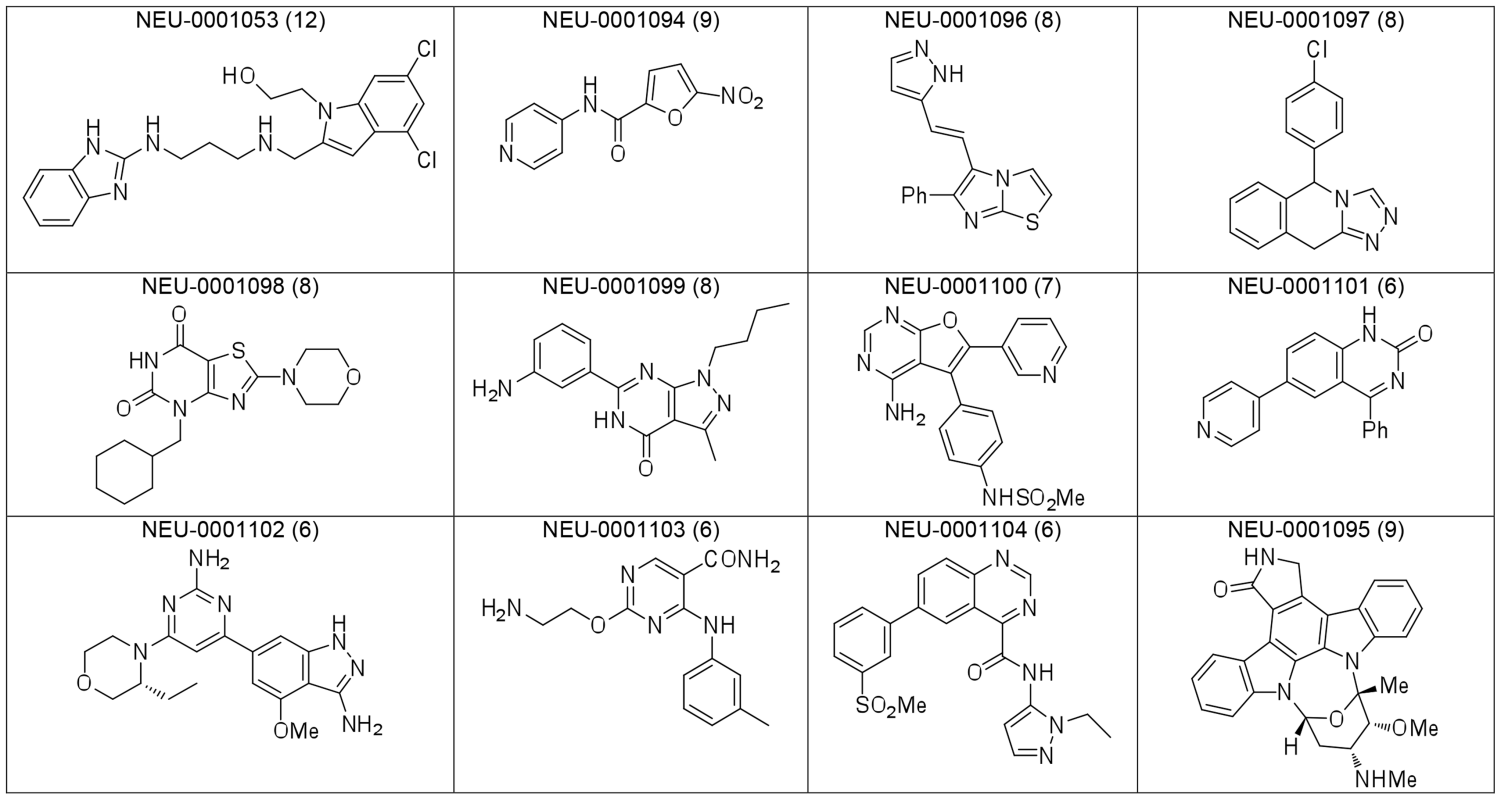
Representative singleton compounds with composite score ≥6. Each compound's composite score is shown in parentheses.

Compounds were visually grouped on the basis of common substructures, which resulted in the delineation of 59 clusters. Of these clusters, 46 clusters had at least one molecule with a composite score of at least 6. **Figures 7** and **8** shows the best-scoring representatives of each of these 46 clusters. Structures of the 13 top-scoring singleton compounds are shown in **Figure 9**, and a summary of their measured and computed data is tabulated in the Supporting Information (**Table S2**).

The Supporting Information (**Table S1**) tabulates average values of key molecular properties for each compound cluster, along with metrics regarding the percentage of each cluster that is fast killing and/or cidal. This allows easy sorting of the compound clusters for prioritization of hit-to-lead medicinal chemistry follow-up.

### Kinase selectivity screening

We selected nine of the high-potency cluster representatives shown in **Figures 7** and **8** for assessment against 15 human kinases in order to ascertain information about potential selectivity liabilities. These kinases were chosen for general selectivity screening owing to their broad representation of the human kinome. The results are shown in the Supporting Information (**Table S3**).

### ADME properties screening

In addition to the computed chemical properties that are often predictive of ADME properties, we mined the GSK database for ADME properties for the hit compounds, and generated this data for the most promising hits when it was not available. We tabulate this data, where available, for the 46 cluster representatives highlighted in the Supporting Information, **Table S4**.

### Mouse pharmacokinetic assessments

**Figure 10:**
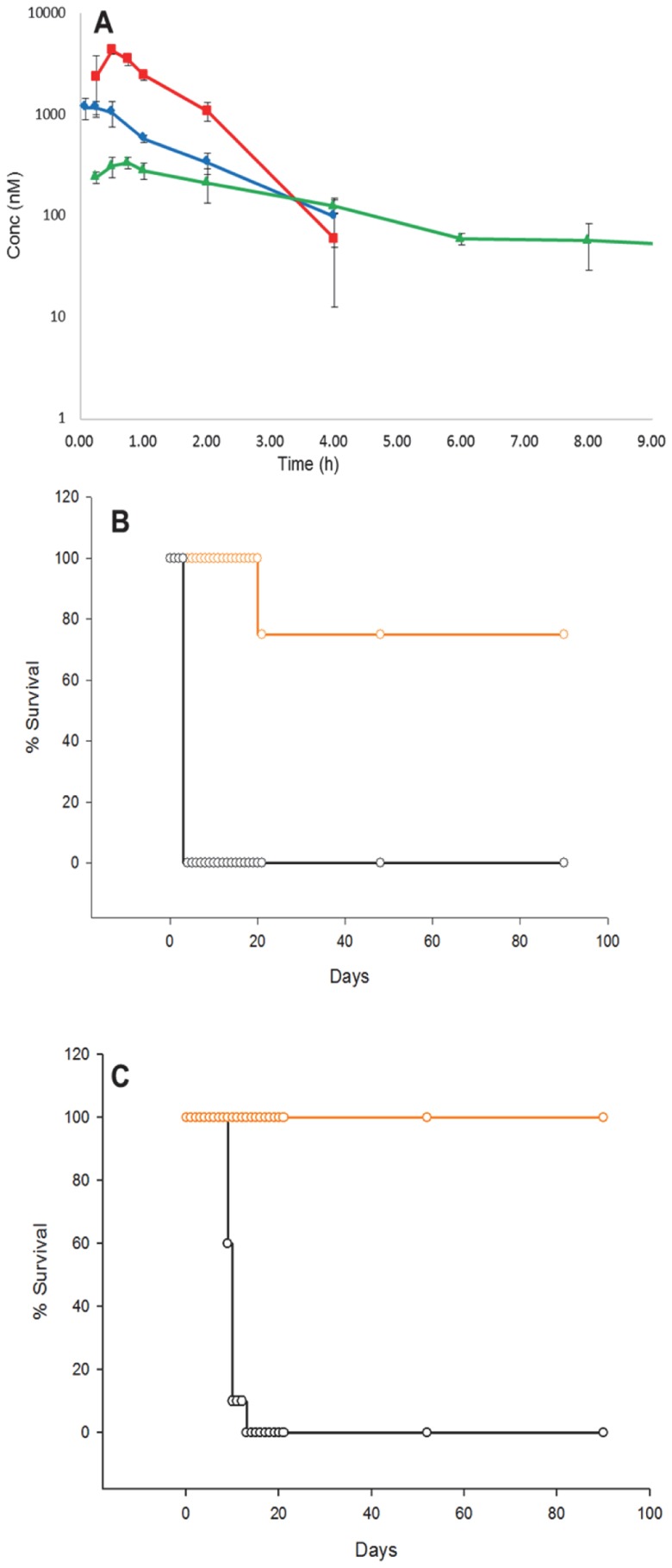
(A) Peripheral blood levels of NEU-1200 (blue), NEU-1207 (red) and NEU-1053 (green) after intraperitoneal administration of 5 mg/kg single dose to NMRI mice (n = 3). The average and standard deviation of the mean for each time point are represented in the plot. Blood levels of NEU-1053 after IP administration was observed till 24h post-dose. Y-axis is represented in log scale. (B) Animal survival plot showing the effects of dosing of NEU-1053 (ip 20/mg/kg/day; orange) versus DMSO control (black line) in *T. b. brucei* or (C) *T. b. rhodesiense* infection. Parasitemia was checked on days indicated by circles.

We selected three of the most attractive hit compounds to be assessed in mouse pharmacokinetic experiments, with an eye towards selecting at least one to test in an *in vivo* model of HAT. We selected NEU-1200 (cluster 32, composite score 13), NEU-1207 (cluster 34, composite score 13), and the top-scoring singleton NEU-1053 (composite score 12). The results of these experiments are shown in **Figure 10A**, and the pharmacokinetic parameters obtained are tabulated in the Supporting Information (**Tables S7**). Of these three, NEU-1053 was selected for assessment in the mouse bloodstream model of HAT on the basis of its combination of high potency (*T. b. brucei* pEC_50_ = 9.17; *T. b. rhodesiense* pEC_50_ = 9.6; *T. b. gambiense* pEC_50_ = 9.7, see **Table S6**), rapid, cidal activity and excellent blood exposure following intraperitoneal (ip) dosing (**Figure 10A**). The total blood exposure was well in excess of the EC_99_ observed for the compound for>24 hours, though we also observed a high plasma protein binding (>99%) in human plasma. This led us to elect to use a higher dose for the efficacy experiments.

### Mouse efficacy model

We focused an *in vivo* efficacy experiment on NEU-1053 on the basis of its excellent PK results and its very high *in vitro* potency. We found this compound to be well-tolerated in mice at i.p. doses of 20 mg/kg. In the bloodstream infection experiments (that mimic Stage 1 HAT in humans), female NMRI mice were infected with *T. b. rhodesiense* (EATRO3 ETat1.2 TREU164) on day 0, and after three days, the infected animals were treated with 10 mg/kg doses of NEU-1053 twice a day for 4 days (days 3–6). Following a four day hiatus, drug was administered for another four days. On day 5, none of the mice treated with NEU-1053 showed any detectable parasitemia (Detection limit: 500 parasites/mL of blood), while the control group showed parasitemia of 15-940 million parasites/mL of blood (Supplementary Information **Table S9**). Control mice succumbed to parasitemia on days 9-13 (80% on days 9–10), while the treated group maintained undetectable parasitemia, except one of them, which had a peak of parasitemia on day 11 (during the second round of treatment).

The rapid clearance of the parasitemia just 24–48 hours after the first treatment suggests NEU-1053 has a potent trypanocidal activity *in vivo* that is consistent with the rate of action and cidality assays described above.

An identical *in vivo* experiment was performed using the highly virulent strain of *T. b. brucei* (Lister 427). In this case, after day 3, none of the mice treated showed any detectable parasitemia, while the control group showed parasitemia of 520–1100 million parasites/mL of blood (Supplementary Information **Table S8**). All the control mice succumbed to parasitemia on day 5 (the third day of the treatment). In contrast, the treated group maintained undetectable parasitemia. After the four day treatment hiatus, parasitemia was detected in 2 out of 4 mice. This second round of treatment resulted again in a reduction of the parasitemia to undetected levels for both positive mice.

We continued to follow parasitemia after the second four-day regimen. One of the previously infected mice died on day 21, with 1.2×10^8^ parasites apparent in the blood on the day prior. However, the three other mice have been deemed to be cured, since 90 days after NEU-1053 treatment the mice are alive and no parasitemia was detected.

In sum, while all the control mice succumbed to the infection, all but one of the drug-treated mice showed parasitological cure for 90 days following the drug treatment.

## Discussion

Based on previous results in testing published investigational human kinase inhibitors against *T. brucei* cultures and mouse infections, we began considering whether a wider exploration of novel kinase inhibitors could provide a more comprehensive set of hit compounds that could afford a broad variety of kinase inhibitor “hits” that could be progressed into new leads for HAT therapeutics discovery.

With this in mind, we selected 42,444 inhibitors from GlaxoSmithKline's compound collection on the basis of their historical relevance to previous and ongoing kinase inhibitor drug discovery programs within the company. In addition, we selected compounds structurally similar to kinase inhibitors that we had previously described to have anti-trypanosomal effects. Notably, the HTS screening set contains the recently-disclosed Published Kinase Inhibitor Set (PKIS) [37], which consists of 367 compounds that GSK has profiled against a wide range of human kinase. We will shortly report the entirety of the *T. brucei* cellular screening data for this subset of the HTS.

The primary assay gave rise to a very large number of compounds that inhibit parasite growth. This is evidence that allows us to infer that perhaps kinase targeted libraries may be biased towards high antiparasitic activity, and, since a significant proportion are most likely operating via trypanosomal kinases, that kinases represent a very powerful class of enzymes to target for growth inhibitors.

The large hit rate we observed provided a unique opportunity to critically evaluate and prioritize the hit compounds on the basis of most desirable properties. We would expect that, based on previous assertions that high-quality hits are most likely to result in to high-quality leads and drugs [38], selecting the best hit compounds from our screen would afford the best opportunity for fruitful lead optimization later. Thus, we elected to prioritize compounds on the basis of not only compound potency and selectivity, but also on the basis of other critical predictors of success for HAT therapeutics discovery, including predicted CNS activity, good physicochemical properties, selectivity over human kinases, as well as desirable cell action properties (rate of action and cidality). These last two points are driven by the expectation that compounds with a rapid and irreversible effect on parasite sells would most likely be effective anti-HAT agents.

Since we have several variables of importance to new HAT lead compounds, we implemented a multivariate Composite Scoring system, similar to that utilized previously for antimalarial [8] and anti-TB HTS hit series [7]. In developing a relative priority scoring system, we have been able to rank order compounds, and clusters of compounds, on the basis of their attractiveness for further study and optimization.

As with any HTS campaign, the compounds identified here will require hit-to-lead and lead optimization medicinal chemistry efforts in order to translate into new clinical candidates. With this in mind, we have provided additional data that allows the research community to prioritize and direct follow-up on these hits. For example, the *in vitro* ADME data shown in the Supporting Information can be directive for identifying and rectifying potential liabilities for a chemotype, such as solubility. Indeed, the inclusion of such data for related compounds can allow particularly useful comparisons.

Many of the compounds tested in this HTS emerged from historical kinase inhibitor programs within GSK. It is not surprising, therefore, that we observe a range of potency values when selected compounds are tested against some human kinases. This data will be useful for future optimization efforts by highlighting potentially important human kinases for which it may be desirable to reduce potency. However, it is not at all clear that absolute selectivity over all human kinases is required (or even achievable). It is possible that inhibition of some human kinases during a short-duration HAT treatment may have little-to-no toxicity effect.

Generally, it is expected that, following an HTS campaign, hit compounds will need extensive optimization in order to achieve efficacy in animal model proof-of-concept. In this case, however, we were able to identify a compound, NEU-1053, which shows profound effects in parasitemia reduction, leading to cure of infection in mice. Based on its CNS MPO score, we expect that this compound is unlikely to penetrate into the CNS (a requirement for any new HAT clinical candidate), yet we believe that this compound represents a powerful endorsement of the whole cell high-throughput screening approach for antiparasitic agents. Work is ongoing to identify potential mechanism(s) of action for this compound, and analog synthesis campaigns are underway to improve physicochemical properties that will enable CNS penetration while maintaining potency.

There are additional, high-potency compounds in the screening set that are currently undergoing PK studies and that, if these results are indicative of good exposure, will be tested in infection models. Also, we continue to look with keen interest in particular at compounds that are predicted to show high CNS exposure; following pharmacokinetic confirmation, such compounds will be advanced into a CNS infection model of HAT. We anticipate reporting such results in due course.

An essential feature of the project spawned at the GSK OpenLab is the open availability of data to the wider neglected disease research community. We have deposited the HTS data shown in the tables within this report, along with human kinase selectivity, physicochemical properties data, and computed properties in a shared data set at www.collaborativedrug.com and in the ChEMBL database. This data is available to anyone who wishes to access it in a searchable format. We encourage collaborative pursuit of these chemical classes as starting points for anti-*T. brucei* agents and we are open to address specific queries on a particular series. In addition, since the kinomes of the trypanosomatid parasites have been shown to be quite similar [19], and since previous reports describe cross-reactivity of compounds across related protozoan pathogens, these compounds may also provide potential starting points for related parasites.

### Supporting information available

Supplementary data tables are included in the Supporting Information as noted throughout the text, annotated with NEU registry numbers to enable online searching within the publically available data set on www.collaborativedrug.com, and in the ChEMBL database.

## Supporting Information

File S1 This file contains additional information regarding the high-throughput screening biological assay development.
(DOCX)

Table S1 Cluster properties.
(DOCX)

Table S2 Properties of the 12 top-scored singleton compounds (from manuscript **Figure 9**).
(DOCX)

Table S3 Human kinase selectivity data for selected top-scored cluster representatives.
(DOCX)

Table S4 Computed and measured ADME properties for cluster representatives.
(DOCX)

Table S5 Percentages of 797 HTS hits in each Composite Scoring bin.
(DOCX)

Table S6 Assessment of compounds against *T. brucei rhodesiense* and *T. brucei gambiense.*
(DOCX)

Table S7 Pharmacokinetic parameters of NEU-1200, NEU-1207, and NEU-1053 obtained in peripheral whole blood after iv, ip, and po administration to NMRI mice. Results are expressed as Mean and Standard Deviation (SD) of n = 3 mice.
(DOCX)

Table S8 Parasitemia counts following treatment of mice infected with *T. b. brucei* with NEU-1053 (20 mg/kg/d).
(DOCX)

Table S9 Parasitemia counts following treatment of mice infected with *T. b. rhodesiense* with NEU-1053 (20 mg/kg/d).
(DOCX)
