# Supplementary material for: Identification and Characterization of Hundreds of Potent and Selective Inhibitors of Trypanosoma brucei Growth from a Kinase-Targeted Library Screening Campaign
Source: PLoS Negl Trop Dis. 2014 Oct 23;8(10):e3253. doi: 10.1371/journal.pntd.0003253 (PMC4207660; doi:10.1371/journal.pntd.0003253)
Supplement: File S1 — This file contains additional information regarding the high-throughput screening biological assay development. (DOCX) [file pntd.0003253.s001.docx]

**Accelerated discovery of multiple high-quality hit series against *Trypanosoma brucei* from a kinase-targeted library*.* An OpenLab initiative.**

Rosario Diaz,^1^ Sandra A. Luengo-Arratta,^1,2^ João D Seixas,^1,2^ Emanuele Amata,^2^ William Devine,^2^ Carlos Cordon-Obras,^1^ Domingo I. Rojas-Barros,^1^ Elena Jimenez,^3^ Fatima Ortega,^3^ Sabrinia Crouch,^3^ Gonzalo Colmenarejo,^3^ Jose Maria Fiandor,^3^ Jose Julio Martin,^3^ Manuela Berlanga,^3^ Silvia Gonzalez,^3^ Pilar Manzano,^3^ Miguel Navarro,^1^* and Michael P Pollastri,^2^*

^1^Instituto de Parasitología y Biomedicina "López-Neyra" Consejo Superior de Investigaciones Cientificas, Granada 18100 Spain. ^2^Department of Chemistry and Chemical Biology and ^3^Center for Drug Discovery, Northeastern University, Boston, MA 02115. ^3^Tres Cantos Medicines Development Campus, DDW and CIB, GlaxoSmithKline, 28760 Tres Cantos, Spain.

**Table of Contents**

| Biological Assay Development | S2 |
| --- | --- |
| References | S16 |

**BIOLOGICAL ASSAY DEVELOPMENT**

***Cell culture.***

Trypanosome cell culture**.** Bloodstream *Trypanosoma brucei brucei* Lister 427 was the selected strain to perform the HTS experiments and the profiling assay. Cell strain was cultured in Hirumi’s modified Iscove’s medium (HMI-9) [[1](#_ENREF_1)], supplemented with 10% heat-inactivated FBS, at 37ºC and 5% CO_2_ in T-25 vented flask (Corning®). For selected hits, compounds were assayed against two pathogenic parasite strains, *Trypanosoma brucei rhodesiense* EATRO3 and *Trypanosoma brucei gambiense* ELIANE: cell lines were grown in HMI-9 supplemented with 20% heat-inactivated FBS, and similar conditions as *T. b.b.* Lister 427.

HepG2 cell culture. HepG2 cell line (human liver hepatocellular carcinoma cell line, ATCC) was cultured in Eagle’s MEM medium supplemented with L-Glutamine, Earle’s Salts, 10% heat-inactivated FBS and 1% non-essential amino acids (NEAA), at 37ºC and 5%CO_2_ in T-175 vented flask (Corning®).

***Activity Assays.***

Assay development.

*Initial cell density and DMSO titration:* 50 μL of increasing concentrations of trypanosoma cells, from 0 to 10,000 cells/mL, were seeded in black, clear-bottom 384-well Greiner microplates containing 0, 100, 200 and 250 nL of DMSO. Plates were covered with lids and incubated for 72 hours at 37 ºC and 5% CO_2_. 2 hours prior ending the incubation, 10 μL of 200 μM Resazurin solution in prewarmed HMI-9 media were added to each well, and plates were incubated two hours more in the same conditions. Generated fluorescence was read in an Envision Multilabel Reader (PerkinElmer) at 535 nm (excitation filter) and 580 mn (emission filter), and obtained data were plotted in SigmaPlot 11.

Potency assessment *in vitro* against *T. b.* *gambiense* and *T. b. rhodesiense*. Assays were performed essentially as described elsewhere [[2](#_ENREF_2)] using bloodstream forms of *T. b. rhodesiense* (strain EATRO3 ETat1.2 TREU164 [[3](#_ENREF_3)]) and *T. b. gambiense* (strain Eliane MHOM/CI/52/ITMAP 2188 [[3](#_ENREF_3)]) maintained in HMI-9 medium (supplemented with 20% inactivated calf foetal serum) at 37ºC in humidified atmosphere containing 5% CO_2_. Briefly, to establish the EC50, cultures of both subspecies were treated with three-fold increasing concentrations of compounds (with similar DMSO increasing concentration as control) in a 96-well plate at a final volume of 100 μl per well. The initial parasite concentration was set at 1x10^3^ and 1x10^4^ parasites per millilitre for *T. b. rhodesiense* and *T. b. gambiense*, respectively. Cell populations were measured at 72 hours with an Infinite F200 microplate reader (Tecan Austria GmbH, Austria); the determination of cell viability was carried out by the established colorimetric technique AlamarBlue^®^, a spectrophotometric assay which measures the ability of living cells to reduce resazurin [[4](#_ENREF_4),[5](#_ENREF_5)]. Pentamidine was used as drug control for potency comparison. The EC50 was defined as the concentration of drug inhibiting 50% of control growth, and was calculated by linear regression analysis using SigmaPlot 10.0 software.

**References.**

1. Hirumi H, Hirumi K (1989) Continuous cultivation of Trypanosoma brucei blood stream forms in a medium containing a low concentration of serum protein without feeder cell layers. J Parasitol 75: 985-989.

2. Diaz-Gonzalez R, Kuhlmann FM, Galan-Rodriguez C, Madeira da Silva L, Saldivia M, et al. (2011) The susceptibility of trypanosomatid pathogens to PI3/mTOR kinase inhibitors affords a new opportunity for drug repurposing. PLoS Negl Trop Dis 5: e1297.

3. Turner CM, McLellan S, Lindergard LA, Bisoni L, Tait A, et al. (2004) Human infectivity trait in Trypanosoma brucei: stability, heritability and relationship to sra expression. Parasitology 129: 445-454.

4. Onyango JD, Burri C, Brun R (2000) An automated biological assay to determine levels of the trypanocidal drug melarsoprol in biological fluids. Acta Trop 74: 95-100.

5. Raz B, Iten M, Grether-Buhler Y, Kaminsky R, Brun R (1997) The Alamar Blue assay to determine drug sensitivity of African trypanosomes (T.b. rhodesiense and T.b. gambiense) in vitro. Acta Trop 68: 139-147.
