# Supplementary material for: Identification and Characterization of Hundreds of Potent and Selective Inhibitors of Trypanosoma brucei Growth from a Kinase-Targeted Library Screening Campaign
Source: PLoS Negl Trop Dis. 2014 Oct 23;8(10):e3253. doi: 10.1371/journal.pntd.0003253 (PMC4207660; doi:10.1371/journal.pntd.0003253)
Supplement: Table S1 — Cluster properties. (DOCX) [file pntd.0003253.s002.docx]

**Table S1**. Cluster properties.

|  | **Average values** | | | | | | | | | | | |  |  |
| --- | --- | --- | --- | --- | --- | --- | --- | --- | --- | --- | --- | --- | --- | --- |
| **Cluster** | **size^a^** | **Score** | **pEC_50_** | **pTC_50_** | **cLogP** | **LE** | **LLE** | **LLEAT** | **TPSA** | **MWt** | **LogD** | **MPO Score** | **% Fast** | **% Cidal** |
| 0 | L | 5.89 | 6.48 | 4.13 | 3.47 | 0.34 | 3.01 | 0.27 | 88.06 | 376.19 | 3.84 | 4.10 | 19% | 4% |
| 1 | S | 5.67 | 6.70 | 4.08 | 3.82 | 0.40 | 2.88 | 0.28 | 85.25 | 329.65 | 4.19 | 4.43 | 0% | 0% |
| 2 | M | 5.00 | 6.72 | 4.46 | 4.18 | 0.30 | 2.54 | 0.23 | 116.84 | 426.37 | 3.47 | 2.67 | 20% | 20% |
| 3 | M | 7.57 | 6.80 | 4.58 | 2.69 | 0.37 | 4.11 | 0.33 | 117.39 | 347.67 | 1.42 | 3.31 | 57% | 29% |
| 4 | M | 6.50 | 6.80 | 4.00 | 2.28 | 0.31 | 4.52 | 0.32 | 106.59 | 432.89 | 3.03 | 4.88 | 17% | 0% |
| 5 | S | 4.00 | 6.33 | 4.08 | 3.94 | 0.22 | 2.39 | 0.19 | 145.83 | 578.58 | 4.04 | 2.79 | 33% | 0% |
| 6 | M | 5.20 | 6.71 | 4.31 | 3.81 | 0.29 | 2.91 | 0.24 | 95.28 | 459.10 | 4.07 | 4.11 | 10% | 0% |
| 7 | S | 6.00 | 6.24 | 4.13 | 2.97 | 0.30 | 3.27 | 0.27 | 94.07 | 379.41 | 3.22 | 4.64 | 50% | 0% |
| 8 | 8 | 4.88 | 6.64 | 4.40 | 3.31 | 0.28 | 3.33 | 0.25 | 109.84 | 463.57 | 3.55 | 2.90 | 13% | 13% |
| 9 | M | 10.69 | 7.60 | 4.78 | 3.89 | 0.42 | 3.70 | 0.32 | 106.00 | 331.80 | 2.91 | 3.94 | 85% | 69% |
| 10 | S | 5.40 | 6.44 | 4.00 | 3.98 | 0.33 | 2.45 | 0.25 | 82.01 | 377.50 | 4.54 | 4.25 | 0% | 20% |
| 11 | S | 6.00 | 6.41 | 3.99 | 3.09 | 0.31 | 3.32 | 0.27 | 100.71 | 393.22 | 3.52 | 4.39 | 20% | 20% |
| 12 | M | 5.60 | 6.37 | 4.06 | 2.98 | 0.30 | 3.39 | 0.28 | 121.01 | 418.39 | 2.93 | 3.77 | 13% | 7% |
| 13 | S | 6.00 | 6.18 | 4.00 | 2.62 | 0.34 | 3.56 | 0.31 | 93.78 | 335.86 | 3.23 | 5.33 | 0% | 0% |
| 14 | L | 7.11 | 6.84 | 4.20 | 3.46 | 0.33 | 3.37 | 0.28 | 98.72 | 395.14 | 3.79 | 3.68 | 57% | 26% |
| 15 | L | 6.98 | 6.90 | 4.26 | 3.31 | 0.36 | 3.60 | 0.30 | 108.66 | 393.75 | 3.51 | 3.43 | 38% | 23% |
| 16 | L | 6.02 | 6.86 | 4.48 | 4.40 | 0.31 | 2.46 | 0.22 | 107.34 | 429.96 | 4.15 | 2.75 | 44% | 33% |
| 17 | L | 8.19 | 6.92 | 4.31 | 3.35 | 0.34 | 3.57 | 0.29 | 98.74 | 385.53 | 3.50 | 4.06 | 69% | 42% |
| 18 | 7 | 5.14 | 6.34 | 4.17 | 2.79 | 0.30 | 3.54 | 0.28 | 100.07 | 407.17 | 2.52 | 4.17 | 0% | 0% |
| 19 | M | 6.10 | 6.43 | 4.19 | 3.78 | 0.34 | 2.65 | 0.25 | 96.64 | 416.26 | 4.13 | 3.92 | 20% | 10% |
| 20 | L | 6.48 | 7.08 | 4.49 | 3.04 | 0.36 | 4.04 | 0.32 | 85.91 | 375.24 | 2.79 | 4.31 | 4% | 0% |
| 21 | 7 | 5.43 | 6.83 | 4.67 | 3.15 | 0.35 | 3.68 | 0.30 | 85.64 | 372.61 | 2.46 | 4.40 | 0% | 0% |
| 22 | S | 8.50 | 7.69 | 4.57 | 3.75 | 0.37 | 3.95 | 0.30 | 61.92 | 390.01 | 2.88 | 3.65 | 50% | 50% |
| 23 | L | 5.76 | 6.38 | 4.08 | 2.60 | 0.33 | 3.78 | 0.31 | 90.11 | 382.24 | 2.82 | 4.71 | 5% | 0% |
| 24 | S | 11.00 | 6.98 | 4.32 | 1.07 | 0.36 | 5.91 | 0.42 | 126.62 | 373.73 | 1.66 | 4.25 | 67% | 67% |
| 25 | S | 5.75 | 6.20 | 4.09 | 5.74 | 0.34 | 0.45 | 0.14 | 57.87 | 477.35 | 6.30 | 2.74 | 75% | 0% |
| 26 | L | 6.07 | 6.68 | 4.19 | 3.29 | 0.37 | 3.38 | 0.30 | 99.36 | 350.48 | 3.27 | 3.83 | 10% | 10% |
| 27 | S | 7.00 | 7.14 | 4.02 | 4.56 | 0.43 | 2.58 | 0.26 | 70.67 | 308.38 | 5.54 | 3.72 | 0% | 0% |
| 28 | M | 7.18 | 6.26 | 4.05 | 1.74 | 0.31 | 4.53 | 0.33 | 104.23 | 405.67 | 2.77 | 4.50 | 55% | 9% |
| 29 | S | 5.80 | 6.51 | 4.02 | 2.83 | 0.31 | 3.68 | 0.28 | 61.50 | 386.46 | 4.67 | 5.21 | 0% | 0% |
| 30 | M | 5.36 | 6.42 | 4.08 | 4.78 | 0.36 | 1.64 | 0.20 | 79.35 | 356.49 | 5.69 | 3.54 | 9% | 0% |
| 31 | M | 5.50 | 6.70 | 4.24 | 3.57 | 0.33 | 3.13 | 0.27 | 84.40 | 397.99 | 4.68 | 4.33 | 0% | 0% |
| 32 | L | 6.43 | 6.60 | 4.16 | 3.39 | 0.32 | 3.21 | 0.27 | 85.34 | 402.79 | 2.80 | 3.85 | 52% | 13% |
| 33 | S | 8.50 | 6.70 | 4.36 | 3.61 | 0.34 | 3.09 | 0.27 | 63.25 | 388.40 | 4.69 | 4.38 | 75% | 50% |
| 34 | M | 5.43 | 6.42 | 4.15 | 4.22 | 0.29 | 2.19 | 0.21 | 80.83 | 424.26 | 4.32 | 3.49 | 29% | 14% |
| 35 | L | 5.96 | 6.80 | 4.50 | 4.63 | 0.27 | 2.17 | 0.20 | 96.43 | 496.99 | 4.26 | 3.06 | 50% | 46% |
| 36 | S | 8.75 | 7.63 | 4.91 | 5.13 | 0.30 | 2.50 | 0.21 | 92.40 | 464.76 | 5.01 | 3.12 | 75% | 75% |
| 37 | S | 6.67 | 6.41 | 4.24 | 2.89 | 0.36 | 3.52 | 0.30 | 87.24 | 328.68 | 3.17 | 5.08 | 33% | 0% |
| 38 | S | 6.00 | 6.36 | 4.22 | 2.82 | 0.38 | 3.53 | 0.32 | 83.48 | 323.16 | 3.67 | 5.18 | 0% | 0% |
| 39 | S | 6.00 | 6.50 | 4.11 | 3.74 | 0.36 | 2.77 | 0.26 | 75.98 | 343.20 | 4.40 | 4.72 | 33% | 0% |
| 40 | S | 5.75 | 6.23 | 4.03 | 2.78 | 0.43 | 3.45 | 0.35 | 94.71 | 287.83 | 3.66 | 4.74 | 0% | 0% |
| 41 | L | 5.25 | 6.88 | 4.13 | 4.70 | 0.34 | 2.18 | 0.22 | 92.71 | 384.87 | 4.83 | 3.40 | 3% | 0% |
| 42 | S | 7.20 | 6.58 | 4.12 | 2.48 | 0.41 | 4.10 | 0.37 | 89.45 | 332.21 | 3.12 | 4.67 | 20% | 0% |
| 43 | L | 5.57 | 6.70 | 4.24 | 2.88 | 0.31 | 3.81 | 0.29 | 137.67 | 420.28 | 2.42 | 3.21 | 4% | 0% |
| 44 | M | 5.80 | 6.39 | 4.11 | 3.17 | 0.38 | 3.23 | 0.30 | 83.41 | 333.14 | 3.29 | 4.58 | 10% | 0% |
| 45 | M | 4.86 | 6.34 | 4.07 | 3.53 | 0.29 | 2.81 | 0.25 | 77.31 | 420.03 | 3.73 | 3.60 | 0% | 0% |
| 46 | S | 5.00 | 6.52 | 4.10 | 3.64 | 0.31 | 2.88 | 0.25 | 101.05 | 455.64 | 4.48 | 3.87 | 0% | 0% |
| 47 | S | 3.33 | 6.32 | 4.05 | 4.84 | 0.26 | 1.47 | 0.18 | 77.37 | 460.12 | 5.20 | 2.80 | 0% | 0% |
| 48 | S | 5.00 | 6.42 | 4.00 | 3.32 | 0.38 | 3.11 | 0.29 | 106.13 | 347.59 | 3.42 | 4.06 | 0% | 0% |
| 49 | S | 5.00 | 6.29 | 4.11 | 4.33 | 0.34 | 1.96 | 0.21 | 99.23 | 363.46 | 4.53 | 3.58 | 0% | 0% |
| 50 | S | 3.50 | 6.34 | 4.14 | 4.39 | 0.24 | 1.95 | 0.19 | 129.62 | 526.20 | 4.14 | 3.13 | 0% | 0% |
| 51 | S | 4.33 | 6.41 | 4.00 | 3.40 | 0.26 | 3.01 | 0.24 | 103.74 | 466.84 | 3.72 | 4.27 | 0% | 0% |
| 52 | S | 3.60 | 6.55 | 3.90 | 5.49 | 0.28 | 1.06 | 0.16 | 76.80 | 453.81 | 5.86 | 2.76 | 0% | 0% |
| 53 | S | 4.50 | 6.38 | 4.04 | 3.60 | 0.31 | 2.78 | 0.25 | 121.74 | 388.23 | 3.76 | 3.47 | 0% | 0% |
| 54 | S | 4.67 | 6.62 | 4.43 | 4.41 | 0.33 | 2.21 | 0.22 | 93.48 | 378.79 | 4.20 | 4.06 | 0% | 0% |
| 55 | M | 5.00 | 6.30 | 4.00 | 3.92 | 0.35 | 2.38 | 0.24 | 75.18 | 332.08 | 5.04 | 4.35 | 0% | 0% |
| 56 | M | 3.71 | 6.24 | 4.13 | 4.79 | 0.27 | 1.45 | 0.18 | 79.06 | 476.60 | 5.02 | 3.40 | 0% | 0% |
| 57 | S | 4.50 | 6.08 | 4.00 | 4.24 | 0.26 | 1.85 | 0.19 | 98.09 | 434.51 | 3.23 | 3.21 | 50% | 0% |
| 58 | S | 5.00 | 6.63 | 4.00 | 5.05 | 0.36 | 1.58 | 0.20 | 61.21 | 339.39 | 5.54 | 3.80 | 0% | 0% |
| 59 | S | 5.00 | 6.26 | 4.00 | 3.27 | 0.32 | 2.99 | 0.26 | 96.90 | 360.39 | 3.41 | 4.19 | 0% | 0% |

^a^Cluster size categorized: Small (1-5 members), Medium (6-19 members), Large (>20 members).
