# Supplementary material for: Identification and Characterization of Hundreds of Potent and Selective Inhibitors of Trypanosoma brucei Growth from a Kinase-Targeted Library Screening Campaign
Source: PLoS Negl Trop Dis. 2014 Oct 23;8(10):e3253. doi: 10.1371/journal.pntd.0003253 (PMC4207660; doi:10.1371/journal.pntd.0003253)
Supplement: Table S2 — Properties of the 12 top-scored singleton compounds (from manuscript Figure 9 ). (DOCX) [file pntd.0003253.s003.docx]

**Table S2**. Properties of the 12 top-scored singleton compounds (from manuscript **Table 4**).

| Compound | **Score** | **pEC50** | **pTC50** | **cLogP** | **LE** | **LLE** | **LLEAT** | **TPSA** | **MWt** | **LogD** | **MPO Score** | **Fast?** | **Cidal?** |
| --- | --- | --- | --- | --- | --- | --- | --- | --- | --- | --- | --- | --- | --- |
| NEU-0001053 | 12 | 9.17 | 4.84 | 5.03 | 0.43 | 4.14 | 0.3 | 77.9 | 432.35 | 3.07 | 2.48 | Y | Y |
| NEU-0001094 | 9 | 6.25 | 4.23 | 1.05 | 0.5 | 5.2 | 0.53 | 100.95 | 233.18 | 1.64 | 5.53 | N | N |
| NEU-0001095 | 9 | 8.04 | 5.29 | 4.19 | 0.31 | 3.85 | 0.26 | 69.45 | 466.53 | 3.13 | 3.14 | Y | nd^a^ |
| NEU-0001096 | 8 | 6.13 | <4 | 4.06 | 0.4 | 2.07 | 0.24 | 45.98 | 292.36 | 4.98 | 4.47 | Y | N |
| NEU-0001097 | 8 | 6.34 | <4 | 2.87 | 0.43 | 3.47 | 0.35 | 30.71 | 281.74 | 5.35 | 4.93 | Y | N |
| NEU-0001098 | 8 | 6.44 | <4 | 2.5 | 0.37 | 3.94 | 0.33 | 80.22 | 350.44 | 4.58 | 5.42 | Y | nd |
| NEU-0001099 | 8 | 6.40 | <4 | 1.74 | 0.4 | 4.66 | 0.4 | 89.59 | 297.36 | 3.29 | 5.5 | N | nd |
| NEU-0001100 | 7 | 6.16 | <4 | 0.93 | 0.31 | 5.22 | 0.37 | 124 | 381.41 | 1.72 | 4.92 | N | nd |
| NEU-0001101 | 6 | 6.08 | <4 | 2.25 | 0.36 | 3.83 | 0.34 | 58.64 | 299.33 | 3.86 | 5.08 | N | N |
| NEU-0001102 | 6 | 6.35 | <4 | 2.26 | 0.32 | 4.09 | 0.32 | 128.2 | 369.42 | 2.62 | 4.31 | N | nd |
| NEU-0001103 | 6 | 6.47 | 4.39 | 2.66 | 0.42 | 3.8 | 0.36 | 116.15 | 287.32 | 2.05 | 3.73 | N | nd |
| NEU-0001104 | 6 | 6.60 | <4 | 1.79 | 0.3 | 4.81 | 0.33 | 106.84 | 421.47 | 2.82 | 4.97 | N | nd |

^a^ nd=not determined.
