# Supplementary material for: Identification and Characterization of Hundreds of Potent and Selective Inhibitors of Trypanosoma brucei Growth from a Kinase-Targeted Library Screening Campaign
Source: PLoS Negl Trop Dis. 2014 Oct 23;8(10):e3253. doi: 10.1371/journal.pntd.0003253 (PMC4207660; doi:10.1371/journal.pntd.0003253)
Supplement: Table S3 — Human kinase selectivity data for selected top-scored cluster representatives. (DOCX) [file pntd.0003253.s004.docx]

**Table S3**. Human kinase selectivity data for selected top-scored cluster representatives.

|  |  |  |  | **pIC50 values** | | | | | | | | | | | | | | |
| --- | --- | --- | --- | --- | --- | --- | --- | --- | --- | --- | --- | --- | --- | --- | --- | --- | --- | --- |
| **Compound** | **Cluster** | **T brucei pEC_50_** | **HepG2 pTC5_0_** | **IKK1** | **SYK** | **P38A** | **JNK1** | **ITK** | **LCK** | **BTK** | **IKK2** | **ROCK1** | **AURB** | **PI3KA** | **JAK3** | **JAK2** | **EGFR** | **LRRK2** |
| NEU-0001106 | 1 | 7.4 | 4.0 | 5.2 | 7.3 | 4.7 | 6.1 | 7.7 | 6.0 | 6.0 | 5.0 | 5.2 | 7.4 | <4.6 | 6.8 | 7.3 | 5.6 | 7.1 |
| NEU-0001127 | 14 | 7.5 | 4.2 | 4.7 | 6.0 | <4.6 | 6.0 | 6.0 | 6.0 | 5.3 | <4.6 | 4.7 | 6.8 | <4.6 | 6.2 | 6.4 | <4.6 | 5.4 |
| NEU-0001142 | 17 | 7.7 | 4.0 | 6.2 | 6.3 | 4.6 | 6.7 | <4.6 | 5.6 | 4.7 | 6.3 | <4.6 | 7.2 | <4.6 | 6.2 | 7.3 | <4.6 | 7.4 |
| NEU-0001240 | 22 | 9.1 | 4.9 | 5.8 | 4.7 | <4.6 | <4.6 | 6.5 | 5.8 | 6.1 | 6.3 | 8.2 | 9.3 | <4.6 | 6.3 | 6.3 | <4.6 | 6.5 |
| NEU-0001187 | 26 | 7.1 | 4.0 | 5.8 | 7.2 | <4.6 | 6.3 | 7.2 | 6.7 | 6.7 | 5.4 | 5.9 | 8.1 | <4.6 | 8.6 | 8.4 | 5.9 | 7.8 |
| NEU-0001191 | 27 | 7.1 | 4.0 | <4.6 | 5.6 | <4.6 | 5.7 | <4.6 | <4.6 | <4.6 | <4.6 | <4.6 | 6.8 | <4.6 | 6.7 | 6.6 | <4.6 | 7.2 |
| NEU-0001206 | 33 | 7.0 | 4.7 | <4.6 | 6.6 | <4.6 | <4.6 | 7.9 | 7.3 | 6.5 | <4.6 | 4.9 | 7.4 | <4.6 | 6.3 | 6.3 | 5.6 | 6.9 |
| NEU-0001207 | 34 | 7.0 | 4.5 | 6.8 | 5.5 | <4.6 | 6.3 | 6.0 | 5.6 | <4.6 | 5.7 | 6.1 | 5.9 | 5.4 | 6.2 | 6.1 | 5.3 | 7.1 |
| NEU-0001225 | 42 | 6.9 | 4.4 | <4.6 | <4.6 | <4.6 | <4.6 | <4.6 | 5.1 | <4.6 | <4.6 | 6.2 | 4.8 | <4.6 | <4.6 | 4.7 | 4.8 | 4.7 |
