# Supplementary material for: Identification and Characterization of Hundreds of Potent and Selective Inhibitors of Trypanosoma brucei Growth from a Kinase-Targeted Library Screening Campaign
Source: PLoS Negl Trop Dis. 2014 Oct 23;8(10):e3253. doi: 10.1371/journal.pntd.0003253 (PMC4207660; doi:10.1371/journal.pntd.0003253)
Supplement: Table S4 — Computed and measured ADME properties for cluster representatives. (DOCX) [file pntd.0003253.s005.docx]

**Table S4.** Computed and measured ADME properties for cluster representatives.

|  |  |  |  |  | **Computed** | | | | | | | | |  | **Measured** | | | | |
| --- | --- | --- | --- | --- | --- | --- | --- | --- | --- | --- | --- | --- | --- | --- | --- | --- | --- | --- | --- |
| **Cmpd** | **Cluster** | **Score** | **Tbb pEC50** | **HepG2 pTC50** | **MPO Score** | **ClogP** | **nHeavy** | **LE** | **LLE** | **LLEAT** | **tpsa** | **mw** | **ChromLogD** | **PFI** | **permeability (nm/s)** | **Aq Sol (uM)** | **HSA % binding** | **CYP3A4VR pXC50** | **CYP3A4VG pXC50** |
| NEU-0001106 | 1 | 7 | 7.4 | <4 | 4.54 | 3.86 | 21 | 0.49 | 3.59 | 0.34 | 78.52 | 314.29 | 3.95 | 6.95 | 280 | 327 | 95.72 |  |  |
| NEU-0001107 | 2 | 7 | 6.4 | <4 | 4.25 | 1.26 | 25 | 0.35 | 5.15 | 0.39 | 141.81 | 359.41 | 1.53 | 4.53 |  | ≥460 | 92.96 |  |  |
| NEU-0001109 | 3 | 11 | 7.2 | 4.9 | 3.54 | 3.31 | 24 | 0.41 | 3.87 | 0.33 | 104.96 | 330.36 | 1.57 | 3.57 | 160 | 228 | 93.61 | <4.3 | 4.6 |
| NEU-0001111 | 4 | 8 | 6.9 | <4 | 5.24 | 1.41 | 24 | 0.39 | 5.49 | 0.42 | 103.18 | 322.36 | 2.37 | 5.37 | 160 | ≥540 | 82.4 | <4.3 | <4.3 |
| NEU-0001112 | 5 | 6 | 6.3 | 5.1* | 3.24 | 4.1 | 33 | 0.26 | 2.24 | 0.2 | 130.99 | 467.45 | 4.09 | 8.09 | 350 | 21 | 96.63 |  |  |
| NEU-0001113 | 6 | 7 | 6.4 | 4.4 | 5.1 | 2.26 | 25 | 0.35 | 4.1 | 0.33 | 101.73 | 350.39 | 2.54 | 6.54 | 16 | 35 | 95.92 |  |  |
| NEU-0001114 | 7 | 8 | 6.2 | <4 | 4.97 | 2.09 | 27 | 0.31 | 4.07 | 0.32 | 103.79 | 359.38 | 2.2 | 6.2 | 380 | 4 |  | 5.1 | 5 |
| NEU-0001115 | 8 | 10 | 7.4 | 5.3 | 3.31 | 4.06 | 30 | 0.34 | 3.3 | 0.26 | 82.96 | 417.53 | 4.4 | 8.4 |  | 2 | 96.86 | 8.1 | 7.6 |
| NEU-0001117 | 9 | 14 | 8.1 | 5.1 | 4.4 | 2.24 | 27 | 0.41 | 5.81 | 0.4 | 107.01 | 360.37 | 2.73 | 5.73 | 280 | 17 | 97.64 | <4.3 | 5.3 |
| NEU-0001120 | 10 | 6 | 6.9 | <4 | 5.12 | 2.97 | 25 | 0.38 | 3.9 | 0.32 | 93.79 | 347.39 | 3.25 | 7.25 |  |  |  |  |  |
| NEU-0001121 | 11 | 9 | 7.2 | 4.9 | 3.34 | 4.07 | 36 | 0.27 | 3.09 | 0.23 | 97.88 | 479.58 | 3.61 | 8.61 | 240 | 2 |  | 5.1 | 5.5 |
| NEU-0001122 | 12 | 12 | 7.0 | <4 | 3.38 | 1.92 | 28 | 0.34 | 5.08 | 0.36 | 119.23 | 399.47 | 0.78 | 3.78 | <3 | 81 |  |  |  |
| NEU-0001125 | 13 | 6 | 6.0 | <4 | 5.12 | 2.65 | 24 | 0.34 | 3.35 | 0.3 | 97.45 | 326.35 | 3.34 | 6.34 | 650 | 235 |  |  |  |
| NEU-0001127 | 14 | 10 | 7.5 | 4.2 | 4.68 | 3.33 | 22 | 0.47 | 4.14 | 0.37 | 69.73 | 298.39 | 4.13 | 6.13 | 380 | 265 |  | <4.3 | 5 |
| NEU-0001133 | 15 | 14 | 8.7 | <4 | 3.14 | 2.84 | 30 | 0.4 | 5.88 | 0.38 | 130.31 | 495.39 | 3.55 | 5.55 | 890 | 4 | 91.99 | 4.9 | 5.2 |
| NEU-0001135 | 16 | 11 | 6.9 | <4 | 3.44 | 2.87 | 23 | 0.41 | 4.07 | 0.35 | 121.61 | 329.74 | 2.24 | 5.24 | 200 | 15 |  | 6.1 | 5.5 |
| NEU-0001142 | 17 | 14 | 7.7 | <4 | 5.33 | 2.4 | 25 | 0.42 | 5.32 | 0.4 | 71.95 | 355.39 | 4.11 | 7.11 | 77 | 1 | 96.02 | 5 | 5.6 |
| NEU-0001172 | 18 | 7 | 7.1 | 4.7 | 4.86 | 2.63 | 29 | 0.34 | 4.5 | 0.32 | 90.38 | 409.5 | 2.12 | 5.12 | 11 | 71 | 94.35 | 5 | 4.4 |
| NEU-0001173 | 19 | 12 | 7.1 | <4 | 4.74 | 2.93 | 22 | 0.44 | 4.22 | 0.37 | 110 | 341.82 | 3.15 | 5.15 | 450 | 340 | 92.8 | 4.6 | 4.9 |
| NEU-0001174 | 20 | 9 | 7.5 | 4.6 | 5.07 | 2.48 | 27 | 0.38 | 5.01 | 0.36 | 91.15 | 365.36 | 3.09 | 7.09 | 93 | 170 | 95.5 |  |  |
| NEU-0001239 | 21 | 6 | 6.9 | 4.8 | 4.49 | 2.9 | 27 | 0.35 | 4.04 | 0.31 | 96.69 | 377.46 | 2.23 | 6.23 | 42 | 113 | 96.6 | 5.4 | 5.3 |
| NEU-0001240 | 22 | 12 | 9.1 | 4.9 | 2.82 | 4.67 | 30 | 0.41 | 4.39 | 0.31 | 69.81 | 416.54 | 3.26 | 7.26 | 180 | 107 | 95.5 | 5.8 | 5.9 |
| NEU-0001179 | 23 | 10 | 6.9 | <4 | 5.33 | 1.3 | 26 | 0.36 | 5.62 | 0.41 | 88.16 | 383.44 | 2.64 | 5.64 |  | 16 |  |  | 5.6 |
| NEU-0001183 | 24 | 13 | 7.5 | 5 | 4.18 | 2.28 | 26 | 0.4 | 5.26 | 0.39 | 113.65 | 370.43 | 3.08 | 5.08 |  | 3 |  |  |  |
| NEU-0001186 | 25 | 7 | 6.2 | <4 | 3.28 | 5.09 | 26 | 0.33 | 1.15 | 0.17 | 66.4 | 390.26 | 5.73 | 7.73 | 250 | 334 |  |  |  |
| NEU-0001187 | 26 | 9 | 7.1 | <4 | 4.82 | 2.02 | 22 | 0.44 | 5.04 | 0.42 | 100.52 | 299.33 | 2.43 | 5.43 | 330 | 252 | 85.65 | <4.3 | 5.4 |
| NEU-0001191 | 27 | 7 | 7.1 | <4 | 3.72 | 4.56 | 23 | 0.43 | 2.58 | 0.26 | 70.67 | 308.38 | 5.54 | 8.54 |  | 22 |  | 5.2 | 7.1 |
| NEU-0001192 | 28 | 11 | 6.5 | <4 | 4.69 | 0.75 | 28 | 0.32 | 5.75 | 0.39 | 115.39 | 402.46 | 2.39 | 5.39 | 140 | 59 | 89.2 |  |  |
| NEU-0001128 | 29 | 6 | 6.3 | <4 | 5.59 | 2.84 | 27 | 0.32 | 3.45 | 0.28 | 56.07 | 365.43 | 4.82 | 7.82 | 310 | 202 | 93.59 | <4.3 | 5 |
| NEU-0001158 | 30 | 8 | 6.4 | <4 | 3.3 | 2.29 | 27 | 0.33 | 4.13 | 0.32 | 128.17 | 410.42 | 2.99 | 5.99 | 13 | 230 |  | 4.3 | 5.8 |
| NEU-0001195 | 31 | 8 | 7.3 | 4.5 | 5.25 | 2.8 | 28 | 0.36 | 4.49 | 0.33 | 95.94 | 381.39 | 4.15 | 8.15 |  |  |  |  |  |
| NEU-0001200 | 32 | 13 | 7.7 | 4.3 | 5.15 | 3.21 | 25 | 0.42 | 4.52 | 0.36 | 49.74 | 331.41 | 2.88 | 6.88 | 310 | ≥405 | 95.7 | <4.3 | 5.6 |
| NEU-0001206 | 33 | 10 | 7.0 | 4.7 | 4.35 | 4.02 | 27 | 0.36 | 3.01 | 0.26 | 55.71 | 352.43 | 3.88 | 7.88 | 460 | 17 |  | <4.3 |  |
| NEU-0001207 | 34 | 13 | 7.0 | 4.5 | 5.35 | 2.63 | 23 | 0.42 | 4.41 | 0.37 | 70.29 | 299.33 | 3.84 | 7.84 | 230 | 12 | 95.36 | 4.7 | 5.9 |
| NEU-0001210 | 35 | 10 | 7.2 | <4 | 4.21 | 3.61 | 31 | 0.32 | 3.62 | 0.27 | 89.96 | 410.47 | 3.18 | 8.18 | 160 | ≥326 | 98.24 | 8.2 | 7.5 |
| NEU-0001217 | 36 | 11 | 8.2 | 4.3 | 2.95 | 4.17 | 34 | 0.33 | 4.01 | 0.27 | 128.54 | 449.51 | 4.18 | 9.18 | 980 | 5 | 92.8 | 4.6 | 5.3 |
| NEU-0001189 | 37 | 8 | 6.4 | 4.3 | 5.36 | 2.63 | 25 | 0.35 | 3.79 | 0.32 | 96.81 | 333.34 | 2.64 | 6.64 | 260 | 100 | 75.73 |  |  |
| NEU-0001219 | 38 | 6 | 6.2 | <4 | 5.23 | 2.51 | 22 | 0.38 | 3.66 | 0.34 | 98.22 | 315.35 | 3.27 | 6.27 | 180 | 33 |  |  |  |
| NEU-0001220 | 39 | 7 | 6.6 | 4.2 | 4.9 | 3.42 | 25 | 0.36 | 3.17 | 0.28 | 80.48 | 343.4 | 3.64 | 8.64 | 360 | 33 |  | 7.5 | 6.3 |
| NEU-0001221 | 40 | 7 | 6.0 | <4 | 5.08 | 2.7 | 20 | 0.41 | 3.31 | 0.34 | 84.22 | 293.32 | 3.71 | 5.71 | 170 | 232 |  |  |  |
| NEU-0001223 | 41 | 6 | 7.0 | <4 | 3.3 | 4.02 | 28 | 0.34 | 3 | 0.26 | 109.58 | 386.43 | 3.35 | 8.35 |  |  |  |  |  |
| NEU-0001225 | 42 | 10 | 6.9 | <4 | 5.17 | 2.38 | 17 | 0.56 | 4.56 | 0.48 | 69.39 | 227.27 | 2.6 | 5.6 | 42 | >=359 | 84.7 | <4.3 | <4.3 |
| NEU-0001883 | 43 | 8 | 6.1 | <4 | 3.5 | -0.2 | 25 | 0.33 | 6.26 | 0.45 | 141.98 | 342.36 | -0.09 | 2.91 | <3 | >=226 |  |  |  |
| NEU-0001228 | 44 | 7 | 6.0 | <4 | 5.37 | 1.22 | 21 | 0.39 | 4.79 | 0.42 | 96.45 | 280.28 | 1.64 | 4.64 | 87 | 58 | 68.79 | 4.6 | 4 |
| NEU-0001231 | 45 | 7 | 6.4 | <4 | 4.5 | 1.19 | 25 | 0.35 | 5.21 | 0.39 | 81.65 | 338.38 | 1.12 | 4.12 | <10 |  |  |  |  |
| NEU-0001233 | 46 | 6 | 6.2 | 4.1 | 5.08 | 2.6 | 27 | 0.32 | 3.64 | 0.29 | 101.05 | 422.91 | 3.43 | 6.43 | 18 | ≥308 | 96.57 | 4.6 | 4.5 |

*Compound selectivity margin = 16-fold
