# Supplementary material for: Identification and Characterization of Hundreds of Potent and Selective Inhibitors of Trypanosoma brucei Growth from a Kinase-Targeted Library Screening Campaign
Source: PLoS Negl Trop Dis. 2014 Oct 23;8(10):e3253. doi: 10.1371/journal.pntd.0003253 (PMC4207660; doi:10.1371/journal.pntd.0003253)
Supplement: Table S5 — Percentages of 797 HTS hits in each Composite Scoring bin. (DOCX) [file pntd.0003253.s006.docx]

**Table S5.** Percentages of 797 HTS hits in each Composite Scoring bin.

|  | **# of compounds (% of total)** | | | |
| --- | --- | --- | --- | --- |
|  | **3** | **2** | **1** | **0** |
| **pEC_50_** | ≥8 | 8>x≥7 | 7>x≥6 |  |
|  | 23  (3%) | 192 (24%) | 582  (73%) |  |
| **Rate of action** |  | Fast |  | Slow |
|  |  | 240 (30%) |  | 557 (70%) |
| **MPO Score** | ≥5 | 3≤x<5 | 1≤x<3 | <1 |
|  | 23  (3%) | 192 (24%) | 582 (73%) |  |
| **Cidal**^a^ |  | Yes |  | No/not tested |
|  |  | 137 (17%) |  | 660 (83%) |
| **LE** | ≥0.4 | 0.4>x≥0.3 | <0.3 |  |
|  | 97 (12%) | 488  (61%) | 212 (27%) |  |
| **LLE** | ≥6 | 6>x≥5 | 5>x≥4 | <4 |
|  | 13  (2%) | 48 (6%) | 139 (17%) | 597  (75%) |

^a^Cidal/static values represent percentage of compounds tested in the cidality assays (N=288)
