# Supplementary material for: Identification and Characterization of Hundreds of Potent and Selective Inhibitors of Trypanosoma brucei Growth from a Kinase-Targeted Library Screening Campaign
Source: PLoS Negl Trop Dis. 2014 Oct 23;8(10):e3253. doi: 10.1371/journal.pntd.0003253 (PMC4207660; doi:10.1371/journal.pntd.0003253)
Supplement: Table S6 — Assessment of compounds against T. brucei rhodesiense and T. brucei gambiense. (DOCX) [file pntd.0003253.s007.docx]

**Table S6.** Assessment of compounds against *T. brucei rhodesiense* and *T. brucei gambiense.*

| **Compound** | ***T. b. gambiense* EC_50_ (nM)** | ***T. b. rhodesiense* EC_50_ (nM)** |
| --- | --- | --- |
| NEU-0001127 | 5.5 ± 0.8 | 41.1 ± 2.4 |
| NEU-0001188 | 3.5 ± 0.4 | 28.6 ± 1.5 |
| NEU-0001053 | 0.152 ± 0.013 | 0.26 ± 0.04 |
| NEU-0001200 | 11.69 ± 1.03 | 8.1 ± 0.4 |
| NEU-0001207 | 40.3 ± 6.9 | 72.8 ± 13.7 |
| NEU-0001144 | 7.9 ± 0.9 | 5.1 ± 0.5 |
| NEU-0001182 | 389.8 ± 28.7 | 197.1 ± 27.5 |
| Pentamidine | 1.5 ± 0.7 | 1.8 ± 0.9 |
