# Supplementary material for: Identification and Characterization of Hundreds of Potent and Selective Inhibitors of Trypanosoma brucei Growth from a Kinase-Targeted Library Screening Campaign
Source: PLoS Negl Trop Dis. 2014 Oct 23;8(10):e3253. doi: 10.1371/journal.pntd.0003253 (PMC4207660; doi:10.1371/journal.pntd.0003253)
Supplement: Table S7 — Pharmacokinetic parameters of NEU-1200, NEU-1207, and NEU-1053 obtained in peripheral whole blood after iv, ip, and po administration to NMRI mice. Results are expressed as Mean and Standard Deviation (SD) of n = 3 mice. (DOCX) [file pntd.0003253.s008.docx]

**Table S7**. Pharmacokinetic parameters of NEU-1200, NEU-1207, and NEU-1053 obtained in peripheral whole blood after iv, ip, and po administration to NMRI mice. Results are expressed as Mean and Standard Deviation (SD) of n=3 mice.

| **Compound** | **Route** |  | **Vss** | **Cl** | **AUCinf** | **t1/2** |
| --- | --- | --- | --- | --- | --- | --- |
|  | **(dose)^a^** |  | **(L/kg)** | **(ml/min/kg)** | **(ng*h/ml) or *(nM*hr)*** | **(h)** |
| NEU-1200 | IV | Mean | 9 | 101 | 165.2/*498.4* | 1.1 |
|  | (1mg/kg) | SD | 1.5 | 4.8 | 7.8/*23.5* | 0.4 |
| NEU-1207 | IV | Mean | 3.5 | 120.6 | 143.3/*478.7* | 0.4 |
|  | (0.9mg/kg) | SD | 0.5 | 26.3 | 35.6/*119* | 0.1 |
| NEU-1053 | IV | Mean | 12.8 | 110.2 | 180.6/*417.7* | 1.7 |
|  | (1.12mg/kg) | SD | 2.5 | 35.7 | 52.2/*120.7* | 0.1 |
| **Compound** | **Route** |  | **Tmax** | **Cmax** | **AUCinf** | **%F** |
|  | **(dose)^a^** |  | **(h)** | **(ng/ml) or *nM*** | **(ng*h/ml) or *(nM*hr)*** |  |
| NEU-1207 | PO | Mean | 0.33 | 251/*838.5* | 335.7/*1121.5* | 46.8 |
|  | (4.5mg/kg) | SD | 0.14 | 111.9/*373.8* | 63.1/*210.8* | 8.8 |
| NEU-1053 | PO | Mean | 0.42 | 28.6/*66.2* | 150.9/*349.0^b^* | 8.5 |
|  | (5mg/kg) | SD | 0.14 | 11.6/*26.8* | 53.3/*123.3* | 0.8 |
|  |  |  |  |  |  |  |
| **Compound** | **Route** |  | **Tmax** | **Cmax** | **AUCinf** | **t1/2** |
|  | **(dose)^a^** |  | **(h)** | **(ng/ml) or *nM*** | **(ng*h/ml) or *(nM*hr)*** | **(h)** |
| NEU-1200 | IP | Mean | 0.28 | 393/*1185.8* | 613.9/*1852.4* | 1.2 |
|  | (5mg/kg) | SD | 0.21 | 66.9/*201.9* | 50.2/*151.5* | 0.4 |
| NEU-1207 | IP | Mean | 0.25 | 1300.0/*4343.0* | 1659/*5542.3* | 0.5 |
|  | (4.5mg/kg) | SD | 0 | 108.2/*361.5* | 180.3/*602.3* | 0.1 |
| NEU-1053 | IP | Mean | 0.42 | 146.7/*339.3* | 777.2/*1797.6* | 7.2 |
|  | (5mg/kg) | SD | 0.14 | 20.4/*47.2* | 303.9/*702.9* | 5.5 |

a) Real doses administered. b) %Extrapolation of AUCinf>20%
