# Supplementary material for: Identification and Characterization of Hundreds of Potent and Selective Inhibitors of Trypanosoma brucei Growth from a Kinase-Targeted Library Screening Campaign
Source: PLoS Negl Trop Dis. 2014 Oct 23;8(10):e3253. doi: 10.1371/journal.pntd.0003253 (PMC4207660; doi:10.1371/journal.pntd.0003253)
Supplement: Table S8 — Parasitemia counts following treatment of mice infected with T. b. brucei with NEU-1053 (20 mg/kg/d). (DOCX) [file pntd.0003253.s009.docx]

**Table S8.** Parasitemia counts following treatment of mice infected with *T. b. brucei* with NEU-1053 (20 mg/kg/d)

|  | DMSO (6.3%) | |  |  | NEU-1053 (20mg/kg/day) | | |  |
| --- | --- | --- | --- | --- | --- | --- | --- | --- |
| Day/Mouse** | 1 | 2 | 4 | 5 | 6 | 7 | 8 | 9 |
| 0 | 1.00E+04 | 1.00E+04 | 1.00E+04 | 1.00E+04 | 1.00E+04 | 1.00E+04 | 1.00E+04 | 1.00E+04 |
| *3 | 1.88E+07 | 2.56E+07 | 2.63E+07 | 2.19E+07 | 2.31E+07 | 2.06E+07 | 2.63E+07 | 1.63E+07 |
| *4 | 5.20E+08 | 8.30E+08 | 1.10E+09 | 8.60E+08 | N.D. | N.D. | N.D. | N.D. |
| *5 | Dead | Dead | Dead | Dead | N.D. | N.D. | N.D. | N.D. |
| *6 | --- | --- | --- | --- | N.D. | N.D. | N.D. | N.D. |
| *11 | --- | --- | --- | --- | N.D. | N.D. | 1.25E+06 | N.D. |
| *12 | --- | --- | --- | --- | 1.00E+03 | N.D. | 4.00E+05 | N.D. |
| *13 | --- | --- | --- | --- | N.D. | N.D. | N.D. | N.D. |
| *14 | --- | --- | --- | --- | 1.00E+03 | N.D. | N.D. | N.D. |
| 17 | --- | --- | --- | --- | N.D. | N.D. | 2.00E+03 | N.D. |
| 19 | --- | --- | --- | --- | N.D. | N.D. | 8.13E+06 | N.D. |
| 20 | --- | --- | --- | --- | N.D. | N.D. | 1.28E+08 | N.D. |
| 21 | --- | --- | --- | --- | N.D. | N.D. | Dead | N.D. |
| 47 | --- | --- | --- | --- | N.D. | N.D. | --- | N.D. |
| 90 | --- | --- | --- | --- | Alive | Alive | --- | Alive |

N.D.: Not Detected (< 500 parasites/ml blood); *Treatment day; **Mouse #3 was removed from the study since no infection occurred after inoculation.
