# Supplementary material for: Identification and Characterization of Hundreds of Potent and Selective Inhibitors of Trypanosoma brucei Growth from a Kinase-Targeted Library Screening Campaign
Source: PLoS Negl Trop Dis. 2014 Oct 23;8(10):e3253. doi: 10.1371/journal.pntd.0003253 (PMC4207660; doi:10.1371/journal.pntd.0003253)
Supplement: Table S9 — Parasitemia counts following treatment of mice infected with T. b. rhodesiense with NEU-1053 (20 mg/kg/d). (DOCX) [file pntd.0003253.s010.docx]

**Table S9.** Parasitemia counts following treatment of mice infected with *T. b. rhodesiense* with NEU-1053 (20 mg/kg/d).

|  | NEU-1053 - 20 mg/Kg/day (parasites/mL) | | | | | Control (DMSO) – 6.5% (parasites/mL) | | | | |
| --- | --- | --- | --- | --- | --- | --- | --- | --- | --- | --- |
| Day/Mouse | 1 | 2 | 3 | 4 | 5 | 6 | 7 | 8 | 9 | 10 |
| 0 | 1.00E+04 | 1.00E+04 | 1.00E+04 | 1.00E+04 | 1.00E+04 | 1.00E+04 | 1.00E+04 | 1.00E+04 | 1.00E+04 | 1.00E+04 |
| *3 | 8.75E+06 | 1.50E+05 | 1.00E+06 | 1.50E+07 | 6.25E+05 | 3.13E+06 | 9.38E+06 | 5.00E+04 | 1.80E+06 | 5.38E+06 |
| *4 | 5.00E+02 | 2.00E+04 | 1.50E+04 | N.D. | N.D. | 5.13E+07 | 1.60E+08 | N.D. in Neub | 1.50E+07 | 1.50E+08 |
| *5 | N.D. | N.D. | N.D. | N.D. | N.D. | 2.20E+08 | 9.40E+08 | 1.50E+07 | 1.28E+08 | 2.60E+08 |
| *6 | N.D. | N.D. | N.D. | N.D. | N.D. | 1.25E+08 | 5.77E+08 | 6.63E+07 | 2.38E+08 | 1.28E+08 |
| 9 | --- | --- | --- | --- | --- | --- | Dead | --- | Dead | --- |
| 10 | N.D. | N.D. | N.D. | N.D. | N.D. | --- | --- | 8.75E+06 | --- | 1.90E+9 - Dead |
| *11 | N.D. | N.D. | N.D. | 6.50E+03 | N.D. | --- | --- | 9.25E+07 | --- | --- |
| *12 | N.D. | N.D. | N.D. | N.D. | N.D. | --- | --- | 4.50E+08 | --- | --- |
| *13 | N.D. | N.D. | N.D. | N.D. | N.D. | --- | --- | 1.85E+9 - Dead | --- | --- |
| *14 | N.D. | N.D. | N.D. | N.D. | N.D. | --- | --- | --- | --- | --- |
| 17 | N.D. | N.D. | N.D. | N.D. | N.D. | --- | --- | --- | --- | --- |
| 18 | N.D. | N.D. | N.D. | N.D. | N.D. | --- | --- | --- | --- | --- |
| 19 | N.D. | N.D. | N.D. | N.D. | N.D. | --- | --- | --- | --- | --- |
| 20 | N.D. | N.D. | N.D. | N.D. | N.D. | --- | --- | --- | --- | --- |
| 21 | N.D. | N.D. | N.D. | N.D. | N.D. | --- | --- | --- | --- | --- |
| 52 | N.D. | N.D. | N.D. | N.D. | N.D. | --- | --- | --- | --- | --- |
| 90 | Alive | Alive | Alive | Alive | Alive | --- | --- | --- | --- | --- |

N.D.: Not Detected (< 500 parasites/ml blood); *Treatment day.
